# Developing a 3D B Cell Lymphoma Culture System to Model Antibody Therapy

**DOI:** 10.3389/fimmu.2020.605231

**Published:** 2021-02-08

**Authors:** Russell Foxall, Priyanka Narang, Bridget Glaysher, Elin Hub, Emma Teal, Mark C. Coles, Margaret Ashton-Key, Stephen A. Beers, Mark S. Cragg

**Affiliations:** ^1^ Antibody and Vaccine Group, Centre for Cancer Immunology, School of Cancer Sciences, University of Southampton Faculty of Medicine, Southampton, United Kingdom; ^2^ Centre for Immunology and Infection, University of York, York, United Kingdom; ^3^ Nuffield Department of Orthopedics, Rheumatology and Musculoskeletal Sciences, Kennedy Institute of Rheumatology, University of Oxford, Oxford, United Kingdom; ^4^ Department of Cellular Pathology, Southampton University Hospital Trust, Southampton, United Kingdom

**Keywords:** diffuse large B cell lymphoma, antibody therapy, adipocyte derived stem cell, cancer associated fibroblast, tumor associated macrophage, 3D co-culture model

## Abstract

Diffuse large cell B cell lymphoma (DLBCL) accounts for approximately 30%–40% of all non-Hodgkin lymphoma (NHL) cases. Current first line DLBCL treatment results in long-term remission in more than 60% of cases. However, those patients with primary refractory disease or early relapse exhibit poor prognosis, highlighting a requirement for alternative therapies. Our aim was to develop a novel model of DLBCL that facilitates *in vitro* testing of current and novel therapies by replicating key components of the tumor microenvironment (TME) in a three-dimensional (3D) culture system that would enable primary DLBCL cell survival and study *ex vivo*. The TME is a complex ecosystem, comprising malignant and non-malignant cells, including cancer-associated fibroblasts (CAF) and tumor-associated macrophages (TAM) whose reciprocal crosstalk drives tumor initiation and growth while fostering an immunosuppressive milieu enabling its persistence. The requirement to recapitulate, at least to some degree, this complex, interactive network is exemplified by the rapid cell death of primary DLBCL cells removed from their TME and cultured alone *in vitro*. Building on previously described methodologies to generate lymphoid-like fibroblasts from adipocyte derived stem cells (ADSC), we confirmed lymphocytes, specifically B cells, interacted with this ADSC-derived stroma, in the presence or absence of monocyte-derived macrophages (MDM), in both two-dimensional (2D) cultures and a 3D collagen-based spheroid system. Furthermore, we demonstrated that DLBCL cells cultured in this system interact with its constituent components, resulting in their improved viability as compared to *ex-vivo* 2D monocultures. We then assessed the utility of this system as a platform to study therapeutics in the context of antibody-directed phagocytosis, using rituximab as a model immunotherapeutic antibody. Overall, we describe a novel 3D spheroid co-culture system comprising key components of the DLBCL TME with the potential to serve as a testbed for novel therapeutics, targeting key cellular constituents of the TME, such as CAF and/or TAM.

## Introduction

Diffuse large cell B cell lymphoma (DLBCL) is the most common type of non-Hodgkin lymphoma (NHL); accounting for approximately 30%–40% of cases (1). Classically, it is sub-categorized into germinal-center B cell-like (GCB) and activated B cell-like (ABC) entities, based on cell-of-origin and gene expression profiling (GEP) (2), with ABC DLBCL associated with substantially worse outcomes with current treatments (3). Recently, more detailed genetic analyzes, indicate a third sub-type (type 3/unclassified) (2) suggesting DLBCL is actually a constellation of related, but genetically disparate diseases (4, 5). Current first line treatment for DLBCL involves Immunochemotherapy, with an anti-CD20 monoclonal antibody (mAb), usually rituximab, in combination with cyclophosphamide, doxorubicin, vincristine and prednisolone (R-CHOP) (1) which results in long-term remission in more than 60% of cases. However, in a subset of patients, the prognosis remains poor, highlighting an unmet need for alternative therapeutic approaches (6, 7). Furthermore, despite growing knowledge of the under-pinning mutations and oncogenic drivers in DLBCL, new targeted therapeutics have so far failed to deliver improved clinical outcomes, highlighting a need for better models to study DLBCL.

Our aim was to develop a 3D model that was both able to recreate *in vitro*, to some degree, the DLBCL tumor environment (TME), and provide a suitable platform for the testing of therapeutic agents. It is important to note that the TME is a complex ecosystem comprising a mixture of malignant and non-malignant cells residing within an extracellular matrix (ECM). Regarding lymphomas, the complexity of the TME varies according to type. Scott and Gascoyne (8) proposed three models to describe these differences, with DLBCL falling between the re-education model typified by follicular lymphoma (FL) and the effacement model exemplified by Burkitt lymphoma (BL).

Key cellular components of the TME include cancer-associated fibroblasts (CAF) and tumor-associated macrophages (TAM) whose reciprocal cross-talk, combined with their interaction with other TME constituents, sculpt the natural history of the disease. CAF comprise an heterogeneous cell population originating from a variety of sources (9, 10). The interactions between CAF and other cellular constituents of the TME, mediated *via* chemical and mechanical signaling, are key to tumor establishment and maintenance (9). Under normal conditions, a clear inter-relationship exists between B cells and the fibro-reticular network, (FRN) of secondary lymphoid organs. This is also observed in pathological conditions (11), particularly in lymphoma and during the formation of tertiary lymphoid structures in the context of inflammation (12). In the case of FL, cross talk between tumor cells and cells of the local FRN drives their differentiation into tumor-supporting lymphoid stroma (13). Similarly in DLBCL, malignant cells and non-malignant TME components have been shown to induce cells of the FRN, specifically Fibroblastic Reticular cells (FRC), to adopt a CAF-like phenotype (14, 15). Furthermore, CAF can promote survival of primary lymphoma cells *in-vitro* (14, 16), further highlighting the intimate inter-relationship between CAF and tumor cells in the DLBCL TME. Similarities between CAF and normal lymphoid fibroblasts have also been reported (17), with human tonsil derived primary stromal cell cultures shown to support the survival and proliferation of DLBCL cell lines (18). We selected adipocyte derived stem cells (ADSC) as our source of primary human lymphoid-like fibroblasts, as they have previously been utilized as an *in vitro* model of the lymphoid-stroma polarization associated with follicular lymphoma (FL) (13).

Macrophages are myeloid cells that play key roles in immunity and tissue homeostasis (19). In solid tumors, TAM can originate *via* local proliferation of tissue resident macrophages or from monocytes recruited to it (20, 21). In DLBCL, the origin of TAM remains unclear, although several studies have linked increased circulating monocyte frequencies with poor prognosis (22), suggesting a role for monocytes as TAM precursors. Although an over-simplification, it has been proposed that in established tumors, TAM feature an M2/anti-inflammatory-like phenotype supporting tumor growth and suppressing immune responses; while soluble factors produced by both malignant and non-malignant cells and constituents of the ECM within the TME provide reciprocal support for the TAM [as recently reviewed, (23)]. Of relevance to treatment of DLBCL, it has previously been shown that M2-like macrophages typically feature a lower ratio of activatory:inhibitory (A:I) Fc gamma receptor (FcγR) expression than their pro-inflammatory “M1-like” counterparts (24). Engagement of activatory FcγR on macrophages by mAb such as rituximab is proposed to play a key role in determining their anti-tumor efficacy (25, 26). Therefore, treatments that can increase the A:I FcγR expression ratio have the potential to augment mAb immunotherapy and overcome tumor suppression as we recently demonstrated with STING agonists (24) in mouse models.

Modeling the complex interactions of the TME *in vitro* with primary human material is challenging. Nevertheless, 3D co-culture systems are attractive, allowing the combination of key cellular populations in an environment that can also recreate, to some degree, the *in vivo* spatial inter-relationships. Several different 3D techniques have been developed, each with their own limitations (27, 28). Scaffolding-based systems offer the flexibility of combining pre-selected cell populations in the context of a 3D matrix. Therefore, we elected to develop a scaffold-based system that would allow the combination of human primary cell populations, including fibroblasts, myeloid cells and tumor cells, within a Type I collagen-based 3D extracellular matrix, with the aim of recapitulating a DLBCL-like TME featuring key cell populations implicated in mediating and modulating the activity of anti-CD20 mAb.

Using this system, we demonstrated that normal and malignant human B cells interact with ADSC-derived human lymphoid-like fibroblasts, in the presence or absence of human monocyte-derived macrophages (MDM), in both 2D and 3D spheroid co-cultures. The latter system augmented DLBCL viability and provided a means to assess immune effector assays using therapeutic mAb. Our data indicate this system has the potential to serve as a testbed for novel therapeutics, targeting key cellular constituents of the TME, such as CAF and/or TAM.

## Materials and Methods

### Primary Human Samples

Ethical approval for the use of human tumor samples was obtained by Southampton University Hospitals NHS Trust from Southampton and South West Hampshire Research Ethics Committee (REC reference 10/H0504/32). Diffuse large B cell lymphoma (DLBCL) cells were acquired from the Human Tissue Authority-licensed School of Cancer Sciences tissue bank at the University of Southampton under ethically approved study (REC reference 228/02/t). Peripheral blood mononuclear cells (PBMC) were obtained from anonymized leucocyte cones from the National Blood Service (Southampton U.K.) and processed within 4 h of preparation. Ethical approval for using human leukocyte cones was obtained by the Southampton University Hospitals NHS Trust from the East of Scotland Research Ethics Committee (REC reference 16/ES/0048). Informed consent was provided in accordance with the Declaration of Helsinki for all samples.

### Cell Preparation

PBMC were isolated from leukocyte cones by density gradient centrifugation (Lymphoprep, Axis-Shield, UK). For some experiments, B and T cells were isolated from PBMC using untouched B cell, or Pan-T cell kits, respectively (Miltenyi Biotec, UK). A proportion of PBMC were frozen down in 10% Dimethyl sulfoxide (DMSO) and 90% Fetal Calf serum (FCS, both from Sigma, UK) and stored in liquid nitrogen until required. Frozen PBMC were thawed, washed and counted. Cell viability, determined with trypan blue (Sigma, UK), was typically >90%. Primary human monocyte derived macrophages (MDM) were generated from fresh PBMC as previously described (26). Primary DLBCL samples were thawed, washed, then rested for 1 h at 37°C/5% CO_2_ prior to dead cell removal *via* density gradient centrifugation. Recovered cells were counted and viability determined with trypan blue (typically >95%). StemPro™ Human Adipose-Derived Stem Cells (ADSC) were purchased from Thermofisher scientific, UK and stored in liquid nitrogen until use. ADSC were cultured in MesenPRO RS™ medium containing the provided supplement (MesenPRO, GIBCO™, Thermofisher Scientific, UK) according to the manufacturer’s instructions. Low passage number ADSC were generated for use in subsequent experiments, frozen down and stored in liquid nitrogen until required. TrypLE™ Express enzyme (GIBCO™, Thermofisher Scientific, UK) was used to detach ADSC from flasks/wells (see **Supplementary Table 1** for volume). The number of cells used to seed flasks/plates (obtained from Corning, UK) is provided in **Supplementary Table 1**.

### Cell Staining and Flow Cytometry

Cells were washed and stained with combinations of fluorescently-labeled antibodies (see **Supplementary Table 2** for details of antibody clones and suppliers) on ice for 30 min, washed, fixed (in a 1:10 dilution of red blood cell lysis solution, Biorad, UK), and washed again. Labeled cells were stored at +4°C, protected from light, and run on the indicated flow cytometer within 24 h of staining.

### Flow Cytometry Analysis

Harvested cells were washed, stained with Fluorochrome-labeled antibodies and run on a FACSCANTO II (BD Biosciences, UK). Doublets were excluded using side scatter (SSC) and Forward scatter (FSC) height and width, and single cells gated for further analysis.

ADSC-derived fibroblasts were identified as CD45- single cells; differentiation of ADSC in response to each cytokine alone or in combination was determined by the expression of the lymphoid fibroblast markers podoplanin, vascular cell adhesion molecule 1 (VCAM-1, CD106) and intercellular adhesion molecule 1 (ICAM-1, CD54) relative to untreated ADSC. Results are shown as geometric mean fluorescent intensity (MFI).

All flow cytometry analysis was carried out using the FCS express software package (Version 3 Research Edition, Denovo software, USA).

### Adipocyte-Derived Stem Cell Differentiation

Adipocyte derived stem cells (ADSC) were differentiated into lymphoid-like fibroblasts according to the protocol designed by Mark Coles and Bridget Glaysher (29) and subsequently developed as an *in vitro* model of Follicular Lymphoma (FL)-associated lymphoid-stroma polarization (13). ADSC were thawed and cultured until approximately 90% confluent. Cells were harvested and re-plated in 6wp or 12wp in either MesenPRO alone (untreated ADSC) or MesenPRO supplemented with 50 ng/ml IL-4 (in-house), 10 ng/ml TNF-α (Peprotech, UK) or 50 ng/ml Lymphotoxin-α/β (R&D systems, Biotechne, UK), alone or in combination. Plates were cultured for 6 days at 37°C/5% CO_2_ and refed at day 3. For immunofluorescence studies 1 × 10^4^ ADSC were cultured on 13 mm coverslips (Cellpath, UK) in 24 well-plates (wp) for 5 days in MesenPRO medium with/without cytokines (37°C/5% CO_2_); immunofluorescent staining was carried out, and images acquired as described below.

### Attachment Assay

ADSC were plated in 12wp and cultured for 7 days with/without cytokines. A vial of PBMC was then thawed, washed, counted and viability determined using Trypan blue; only samples with viability ≥80% were used. PBMC were rested for 1 h at 37°C/5% CO_2_ prior to use. ADSC cultures were washed, 2 × 10^6^ PBMC/well added and the plates incubated for 2 h (37°C/5% CO_2_). After incubation the plates were imaged using phase contrast microscopy (4× and 10× magnification, Olympus CKX41). Plates were gently washed, and their contents harvested using TrypLE™ express. Recovered cells were stained with antibodies against lymphoid fibroblast markers and CD45 and samples run on a FACSCANTO II. Doublets were excluded and single cells gated as described above. The proportion of CD45+ cells in the samples from co-culture wells was determined using a histogram plot, with the cut-off set with isotype control antibodies. ADSC differentiation into lymphoid-like fibroblasts was confirmed by analyzing ADSC cultured with or without cytokines (see **Supplementary Figure 3**).

### 2D and 3D Co-Culture Assays

ADSC were thawed, washed and counted. T75 Flasks were seeded with ADSC (**Supplementary Table 1**) and cultured for 3 days (37°C/5% CO_2_). On day −1 ADSC were harvested, washed, resuspended in MesenPRO, plated in 6wp and cultured overnight (37°C/5% CO_2_). On day 0 the various cellular constituents for the co-cultures were prepared (see above) and resuspended in MesenPRO +/− cytokines, to give the required concentrations (see below) and then placed on ice. ADSC cultures were washed once and 10^6^ of the previously prepared lymphocytes/primary DLBCL cells added to give a lymphocyte/DLBCL to ADSC ratio of 100:1. In some instances, increasing numbers of MDM were also added to give MDM to ADSC ratios of 1, 5, or 10:1. Differentiation controls of ADSC alone cultured with MesenPRO +/− cytokines were set up in parallel. On day 3, plates for 2D culture were refed with fresh MesenPRO +/− cytokines as appropriate. In some cases, a well per condition was harvested and the recovered cells stained with combinations of fluorescently labeled antibodies, and samples run on a FACSCANTO II. All wells to be used for 3D co-culture, were harvested using TrypLE™ express, the cells pelleted (400g, 5 min, full brake), all supernatant removed, and the cells placed on ice. Type I collagen (Scientific Laboratory Supplies, UK) and 10× DMEM (Sigma, UK), were mixed on ice; 1M NaOH (Sigma, UK) was added incrementally until the solution turned bright pink (neutral pH). The collagen solution was further diluted with ice cold MesenPRO +/− cytokines to obtain a 1 or 2 mg/ml collagen solution. Working on ice, each cell pellet was resuspended in 120 µl of ice-cold collagen solution and mixed well. Twelve microliters of this cell mixture was transferred to the reverse of a 6wp lid, generating 10 spheroids. The lid was then carefully inverted and placed over the base of a 6wp containing MesenPRO +/− cytokines, as required, and incubated for 30 min at 37°C/5%CO_2_ to allow the collagen to set. Once set, the spheroids were gently lifted with forceps (Fisher Scientific, UK), and transferred to the appropriate well. These plates were transferred to an incubator (37°C/5% CO_2_). On day 4, the 3D collagen spheroid cultures were transferred to new 6wp containing fresh MesenPRO +/− cytokines. 2D and 3D cultures were re-fed on Day 6; in some cases, 10 µg/ml of an irrelevant isotype control (trastuzumab) or rituximab was added to 3D spheroid co-cultures at 44 or 20 h prior to the end of the culture. At the end of the culture, a proportion of spheroids were prepared for either immunofluorescence microscopy or embedding in paraffin (see below). The remaining spheroids were washed 3 times, transferred to microcentrifuge tubes and pulsed briefly (Eppendorf Microfuge, 2000g). The supernatant was discarded and 250 µl/tube of 0.4 mg/ml Liberase TL (Roche, UK), added. Samples were incubated in a shaking heated block (Eppendorf, UK) for 10 min at 37°C. Up to 3 further 5-min incubations were performed, where necessary, to ensure spheroids were completely digested. The reaction was quenched by adding 1 ml of complete RPMI (cRPMI: RPMI supplemented with 2mM Glutamine, 1mM Pyruvate, and 100 IU/ml Penicillin/Streptomycin (all from GIBCO, UK) and 10% FCS). Cells were spun down (Eppendorf microcentrifuge, 5 min, 500g, room temperature) and washed twice in 1 ml of PBS (Severn Biotech Ltd, UK) supplemented with 2mM EDTA (VWR, UK). In some experiments, cells recovered from 2D cultures and 3D collagen spheroids were stained with the eBioscience fixable live/dead stain (Invitrogen, UK) according to the manufacturer’s instructions prior to staining with combinations of fluorescently labeled antibodies. All samples were run on a FACSCANTO II. Doublets were excluded, and single cells gated for further analysis. ADSC-derived fibroblasts were identified as CD45- single cells; the degree of differentiation of ADSC in the various 2D and 3D mono and co-culture conditions was assessed by the expression of the fibroblast markers podoplanin, VCAM-1 and ICAM-1 relative to untreated ADSC. Results are shown as geometric mean fluorescent intensity (MFI). Cell viability was determined as the proportion of cells that were live/dead viability stain negative. For a given experiment, all 2D and 3D mono and co-cultures were set up in parallel, using the same cell constituents. For 2D immunofluorescence studies 1 × 10^6^ primary human B cells or primary DLBCL cells were co-cultured with 1 × 10^4^ ADSC to give a ratio of 100 B cells/primary DLBCL cells: 1 ADSC on coverslips as described above. Immunofluorescent staining and imaging were carried out as described below.

### CFSE-Labeling

Primary DLBCL cells were resuspended at 10 × 10^6^ cells/ml in warm PBS and labeled with 2.5 µM or 5 µM CFSE (Molecular probes, UK) for 15 min at room temperature, protected from light. 5 volumes cRPMI was added and the cells incubated on ice for 5 min. Cells were washed twice in 5 volumes of cRPMI, prior to resuspension at 5.0 x10^6^ cells/ml.

### 2D Antibody-Dependent Cell Phagocytosis (ADCP) Assay

CFSE-labeled primary DLBCL cells were used as the target cell population and MDM as the effector cell population in standard ADCP assays, performed as previously described (30). Briefly, CFSE-labeled primary DLBCL cells were, washed, and opsonized with 10 µg/ml of an irrelevant antibody control (trastuzumab) or rituximab. Opsonized target cells were co-cultured with pre-plated MDM at a target:effector ratio of 5:1 for 60 min (37°C/5% CO_2_). 1.5 µl of an in-house labeled anti-FcγRIII APC mAb (see **Supplementary Table 2**) was then added to the required wells, and the plate incubated at room temperature for 20 min protected from light. The plates were then gently washed, 140 µl of cold PBS added per well, and the plate incubated on ice for 30 min to 1 h, protected from light. Working on ice, each well was scraped, and its contents transferred to a flow cytometry tube (BD Biosciences, UK), prior to running on a FACSCalibur (BD Biosciences, UK). A minimum of 1,000 events/tube were collected. A macrophage gate was drawn according to Forward and Side scatter characteristics and the degree of ADCP determined as the % of FcγRIII+ macrophages co-staining with CFSE.

### 3D ADCP Assay

ADCP in 3D collagen spheroid co-cultures treated with 10 µg/ml of rituximab or isotype control (trastuzumab) was determined as follows. Cell suspensions recovered from 3D co-cultures containing ADSC, CFSE-labeled DLBCL and MDM were stained with an antibody panel including CD45 and CD11b and run on a FACSCanto II. Following doublet exclusion, the proportion of CFSE+ CD11b+ cells within an SSC high CD45+ gate was determined; the CFSE cut off was set using unlabeled DLBCL recovered from spheroids cultured in parallel, while an isotype control was used to set the cut off for CD11b. The proportion of DLBCL cells recovered from mAb-treated spheroids was identified as the percentage of SSC low CD45+ single cells. The expression of CD19, CD20 and FcγRIIB on these cells was assessed by flow cytometry in three experiments. The expression levels of these markers on DLBCL recovered from treated spheroids were normalized relative to those on DLBCL recovered from their untreated counterparts, run in parallel.

### Immunofluorescence Staining

Coverslip mono and co-cultures: coverslips were washed, fixed with 4% paraformaldehyde (PFA (Sigma, UK), prepared in house) for 20 min at room temperature, then washed again. Coverslips were blocked with a 2.5% solution of normal goat serum (NGS, Thermofisher Scientific, UK) in PBS for 2 h at room temperature, or overnight at +4°C. When required, permeabilization with PBS/0.15% Triton X-100 (Sigma, UK) for 10 min, followed by a PBS wash was carried out prior to blocking. After blocking, coverslips were washed and incubated with anti-human VCAM-1 and anti-human podoplanin (see **Supplementary Table 2** for details) for 1 h at room temperature. Samples were then washed and stained with the species-appropriate secondary antibodies (**Supplementary Table 2**) for 45 min, protected from light. Samples were washed blocked in PBS/2.5% normal mouse serum (NMS, Thermofisher Scientific, UK) for 30 min, then stained with directly conjugated anti-human ICAM-1 Alexa 647 (**Supplementary Table 2**) for 1 h at room temperature protected from light. After incubation, coverslips were washed, and stained with DAPI (1µg/ml, Molecular probes, UK) in PBS for 10 min at room temperature protected from light, washed twice more and mounted on slides (2 coverslips/slide) in Vectashield (Vector Labs, UK). Slides were left for 15 min to allow the Vectashield to permeate cells, and then sealed. Slides were stored for no more than 8 days at +4°C, protected from light, prior to imaging on a confocal microscope (Leica SP5, Leica, UK) with a 40× oil immersion objective. Images were acquired and processed using LAS-AF Lite software version 4.0 (Leica UK, free download).

### Whole Mount Staining of 3D Spheroid Cultures

Three to four collagen spheroids per culture condition were fixed in 4% PFA for 20–30 min at room temperature, washed three times with PBS and then stored at 4°C until staining. Prior to staining, one to two spheroids/condition were transferred to a flat-bottomed 96wp (Corning, UK), with all subsequent steps carried out in the plate. Spheroids were permeabilized with PBS/0.15% Triton X-100, washed three times with PBS and then blocked with PBS/2.5% of the appropriate animal serum at room temperature for 2 h protected from light. Following blocking, spheroids were stained with a primary antibody diluted to the appropriate concentration in PBS/2.5% appropriate animal serum/0.15% Triton-X-100 (**Supplementary Table 2**). After overnight incubation at 4°C, protected from light, spheroids were washed 4 times with PBS and stained with the appropriate fluorochrome-labeled secondary antibody diluted in PBS + 2.5% of the appropriate species-specific serum (**Supplementary Table 2**). For CD68 staining a 1:100 dilution of CD68 in PBS/2.5% NGS/0.15% Triton X-100 was added to the spheroid containing wells and incubated overnight at +4°C. Wells were washed and the appropriate secondary antibody, diluted in PBS+2.5% NGS, was added and the plate incubated for 1 h at room temperature, protected from light. Wells were washed with PBS. Where applicable, staining for ICAM-1 was performed after this step. Spheroids were blocked with PBS/2.5% NMS for 30 min, washed and stained with 1:50 dilution of mouse anti-human ICAM-1 Alexa 647 in PBS/2.5% NMS for 1 to 2 h. Following staining, cells were washed 3 times with PBS. Spheroids were counterstained with 10 µg/ml DAPI for 10–15 min, washed with PBS and the spheroids stored in PBS at 4°C until imaged. Spheroids were transferred to ibidi glass bottomed slides (Thistle Scientific, UK) prior to imaging on a confocal microscope (Leica SP5 or SP8, Leica, UK) with a 63× oil immersion objective. Images were acquired and processed using LAS-AF Lite software, version 4.0 (Leica, UK, free download).

### Immunohistochemical Staining

Formaldehyde fixed paraffin embedded (FFPE) sections of normal human tonsil tissues and tissue microarrays (TMA) constructed from archival ABC and GCB DLBCL diagnostic biopsy samples were provided by the Research Histology Unit, University Hospital Southampton NHS Foundation Trust. Several collagen spheroids per culture condition were randomly selected for formalin fixation and subsequent paraffin embedding. Spheroids were washed in PBS and fixed in a 10% formalin solution (Sigma, UK). Paraffin embedding and sectioning was carried out by the Histochemistry Research Unit, University Hospital Southampton NHS Foundation Trust. Three-micrometer sections were cut and transferred to slides. Collagen spheroid and TMA sections were stained by immunohistochemistry (IHC) using the fully automated BOND MAX or BOND RX IHC staining instruments (Leica Biosystems, U.K.) using BOND reagents (Leica Biosystems, U.K.) according to the manufacturer’s instructions. Antibodies were diluted in BOND™ Primary Antibody Diluent (Leica Biosystems, UK). Briefly, sections were deparaffinized, pre-treated for heat-induced Ag retrieval (BOND ER1, or ER2 protocol), and incubated with hydrogen peroxide followed by the indicated antibody. The antibody was subsequently bound to the Poly-HRP IgG reagent before incubation with 3,3′-diaminobenzidine (DAB). The sections were subsequently incubated with the indicated antibody, which was then bound to the Post Primary IgG linker reagent. The substrate chromogen Fast Red was then applied. All sections were counterstained using hematoxylin and mounted in CV Ultra mounting media (Leica Biosystems, UK). Slides were imaged at 4×, 10×, and 40× magnification with an Olympus CKX41 microscope. Single color IHC DAB staining of normal human tonsil tissue sections was carried out to validate antibodies and confirm the expression/co-localization of fibroblast markers in lymphoid tissue (**Supplementary Figure 1**).

### Cluster Count and Average Cluster Size Analysis

Phase contrast images of 2D co-cultures of ADSC and B cells or primary DLBCL cells were taken with an Olympus CKX41 microscope. Cluster numbers and average size were analyzed using ImageJ (31). Briefly, images were converted to binary and a size exclusion of 1,000 pixel units was applied to eliminate small clusters of B/DLBCL cells. The clusters were then manually counted, and their area measured using ImageJ.

### Statistical Analysis

All statistical analyzes were performed using Graphpad Prism Version 8.2.1. Two group comparisons were made with unpaired or paired t tests. One-way analysis of variance (ANOVA) was used to compare the expression of markers on ADSC-derived fibroblasts recovered from 2D and 3D mono and co-cultures. Each experiment utilized a single batch of PBMC/DLBCL and MDM combined with ADSC, thus data sets from a single experiment were treated as paired data. We assumed a Gaussian distribution. If a data set was missing from an experiment, a mixed effects model was applied. The recommended Sidak test for multiple comparisons was applied, to both one-way ANOVA and mixed effects tests. p values <0.05 were considered significant; statistical significance was denoted as follows: *p<0.05, **p<0.01 ***p<0.001 and ****p<0.0001.

## Results

### Primary DLBCL Cells Die *In Vitro*

**Figure 1:**
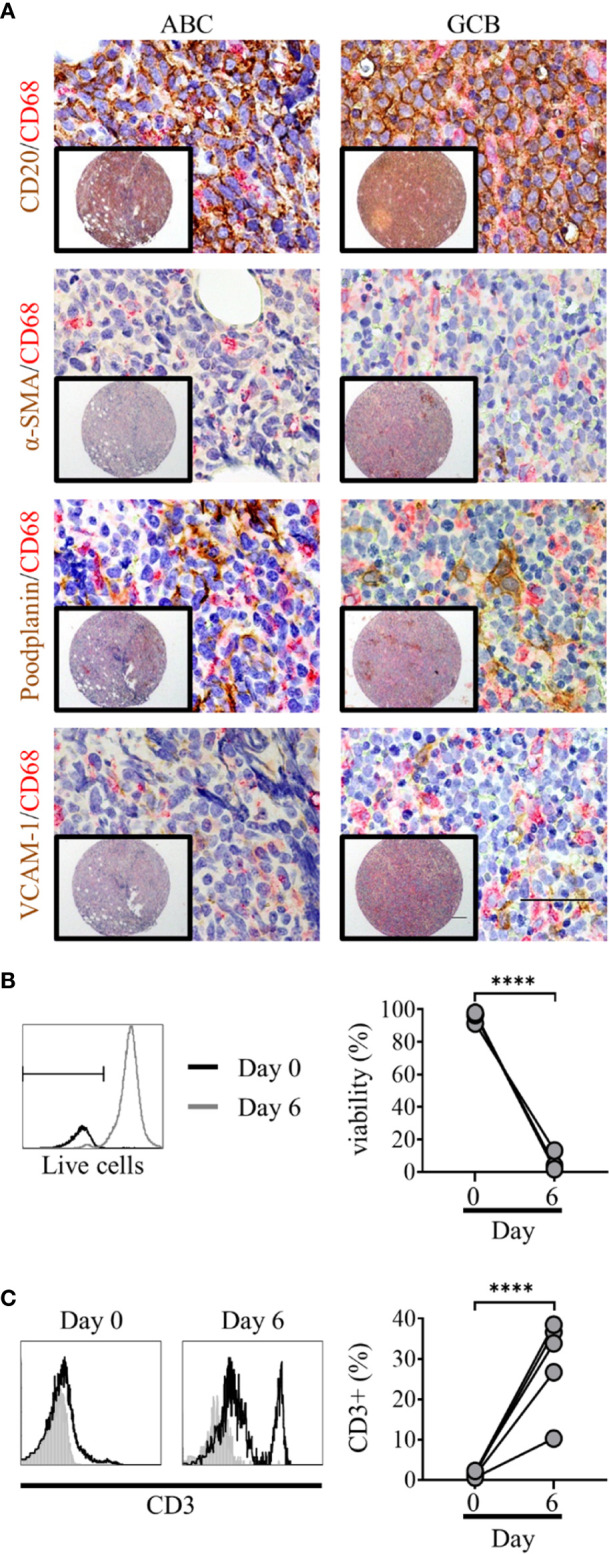
Primary diffuse large cell B cell lymphoma (DLBCL) cells exist within a complex multicellular environment and die *in vitro*. **(A)** Double immunohistochemistry (IHC) staining of DLBCL cells (CD20, brown) or lymphoid fibroblasts [alpha smooth muscle Actin (α-SMA), podoplanin, and VCAM-1 (brown)] and macrophages (CD68, red) of activated B cell-like (ABC) (left hand panels) and germinal-center B cell-like (GCB) (right-hand panels) DLBCL tumor micro-array samples, demonstrating the close interaction of all three cell types within the tumor microenvironment. Scale bars: 200 µm, inset image, 50 µm main image. **(B)** Representative histograms of live/dead viability staining of primary DLBCL cells pre (day 0, black line) and post 6 days of *in vitro* culture (grey line); marker denotes the cut-off for live staining (dead cells stain more intensely). Graph shows pre- and post-culture viability, each filled circle represents an independent experiment. Statistical significance between groups was assessed using a paired two tailed t-tests, ****p < 0.0001. **(C)** Representative histograms showing CD3 staining in primary DLBCL cell suspensions before (day 0) and after 6 days of *in vitro* culture. Graph shows pre- and post-culture percentages of CD3+ lymphocytes, each filled circle represents an independent experiment. Statistical significance between groups was assessed using a paired two tailed t-tests, ****p < 0.0001.

The DLBCL TME is complex, with tumor cells interacting with stroma and immune cells (32). To assess TME cellular architecture in more detail we constructed tissue microarrays (TMA) from archival ABC and GCB DLBCL diagnostic biopsy samples and applied IHC for appropriate cell-specific targets. Prior to this, staining of tonsil tissue was used to validate antibodies and determine the expression/co-localization of fibroblast markers in normal lymphoid tissue (**Supplementary Figure 1**). Both lymphoid fibroblasts, positive for alpha-smooth muscle actin (α-SMA), podoplanin and VCAM-1, and CD20+ DLBCL cells were observed in close proximity to CD68+ macrophages (**Figure 1A**). These observations demonstrate the complex nature of the DLBCL microenvironment and confirm the presence of a heterogeneous network of cell-cell interactions. Next, we assessed the dependence of primary DLBCL cells on their microenvironment for survival. Primary DLBCL were thawed and, following dead cell removal, cultured for 7 days *in vitro*. Over this time, nearly all the DLBCL cells died (viability <2%, **Figure 1B**). In contrast, the relative proportion of CD3+ T cells significantly increased in these cultures, reflecting the specific loss of DLBCL cells during culture (**Figure 1C**). These observations confirm that a more complex *in vitro* multicellular model, better recapitulating the *in vivo* microenvironment, is required to support primary DLBCL cell survival *in vitro*.

### Human Adipocyte-Derived Stem Cells Can Be Differentiated Into Lymphoid Stroma

Human ADSC are multi-potent mesenchymal stem cells, isolated from human adipose tissue, with the capacity to differentiate into different cell types dependent upon their cytokine milieu (33, 34). Data from animal studies indicate a key role for LT-β in driving the differentiation of adipocyte precursors into lymph node stromal cells (35). Similarly, in an *in vitro* model of FL, Pandey et al. (13) showed stimulation of human ADSC with LT-α/β and TNF-α resulted in their differentiation into lymphoid fibroblasts expressing VCAM-1 and ICAM-1, with subsequent addition of IL-4 modulating the phenotype of these cells. Simultaneous treatment of ADSC with all three cytokines has been shown to upregulate cell-surface markers typical of lymphoid fibroblasts, such as α-SMA, podoplanin and the adhesion molecules ICAM-1 and VCAM-1 (29). Therefore, we attempted to develop these cells as a route toward reconstructing the DLBCL TME.

**Figure 2:**
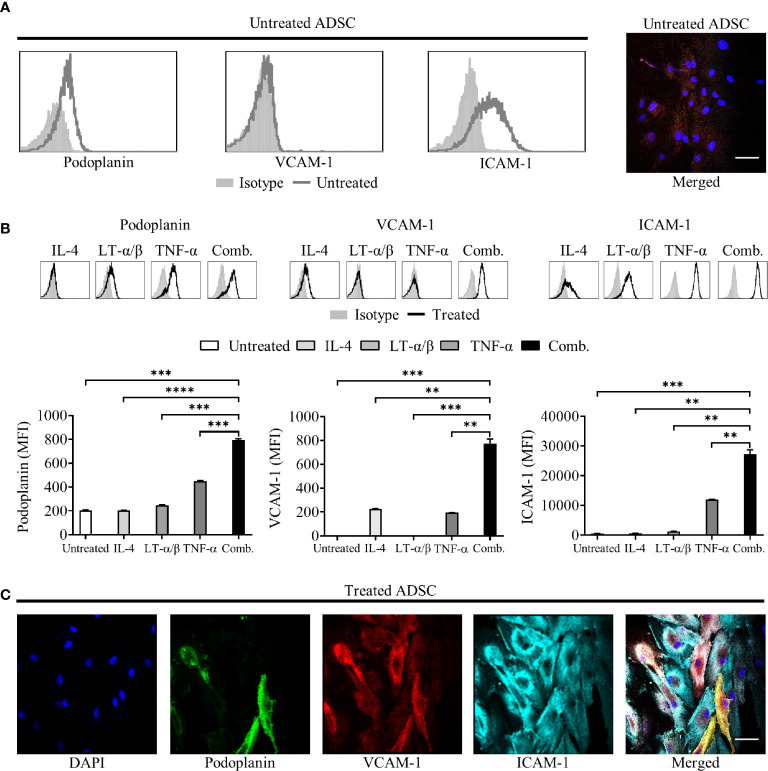
Adipocyte derived stem cells (ADSC) can be differentiated into lymphoid fibroblasts. **(A)** Representative histogram overlays of podoplanin, vascular cell adhesion molecule 1 (VCAM-1) and intercellular adhesion molecule 1 (ICAM-1) expression on untreated ADSC after 6 days of culture, isotype controls are shown as filled silver histograms. Right-hand image depicts immunofluorescent staining of ADSC cultured on coverslips in media alone for 5 days, then stained with fluorescently labeled antibodies to podoplanin (green), VCAM-1 (red) and ICAM-1 (cyan), and counterstained with DAPI nuclear stain (blue); the panel depicts the merged image, scale bar, 50 µm. **(B)** Representative histogram overlays of podoplanin, vascular cell adhesion molecule 1 (VCAM-1) and intercellular adhesion molecule 1 (ICAM-1) expression on ADSC after 6 days of culture with IL-4, LT-α/β, or TNF-α alone, or in combination, isotype controls are shown as filled silver histograms. Graphs show surface expression of ICAM-1, VCAM-1 and podoplanin [Geomean Fluorescence intensity (MFI)] on ADSC cultured under the indicated conditions at day 6 of culture. The mean +/− SD are shown for three independent experiments with each condition performed in triplicate. Statistical significance between groups was assessed using a paired two tailed t-tests, **p < 0.01 ***p < 0.001, and ****p < 0.0001. **(C)** Images depict immunofluorescent staining of ADSC cultured on coverslips in media supplemented with IL-4, LT-α/β, and TNF-α for 5 days, then stained with fluorescently labeled antibodies against the indicated markers, and counterstained with DAPI; panels depict each channel alone, with the final panel showing the merged channels, scale bar, 50 µm.

Under standard culture conditions, ADSC expressed low levels of podoplanin and ICAM-1 and lacked expression of VCAM-1, as confirmed by Flow cytometry and IF microscopy (**Figure 2A**). Treatment of ADSC with LT-α/β, TNF-α, and IL-4 alone or in combination confirmed that all three cytokines were required to drive differentiation into lymphoid-like fibroblasts, as indicated by increased podoplanin and ICAM-1 expression and induction of VCAM-1 expression (**Figure 2B**). Immunofluorescence microscopy of ADSC treated with the cytokine combination confirmed our flow cytometry data (**Figure 2C**). We also demonstrated that these cells are highly dynamic with respect to their differentiation, whereby removal of cytokines resulted in their de-differentiation with a phenotypic switch back to untreated ADSC. Conversely, re-exposure to cytokines resulted in re-expression of the phenotypic markers akin to the original cytokine treated ADSC (**Supplementary Figure 2**), highlighting the dynamic nature of these cells.

### ADSC-Derived Fibroblasts Interact With PBMC in Standard 2D *In Vitro* Cultures

**Figure 3:**
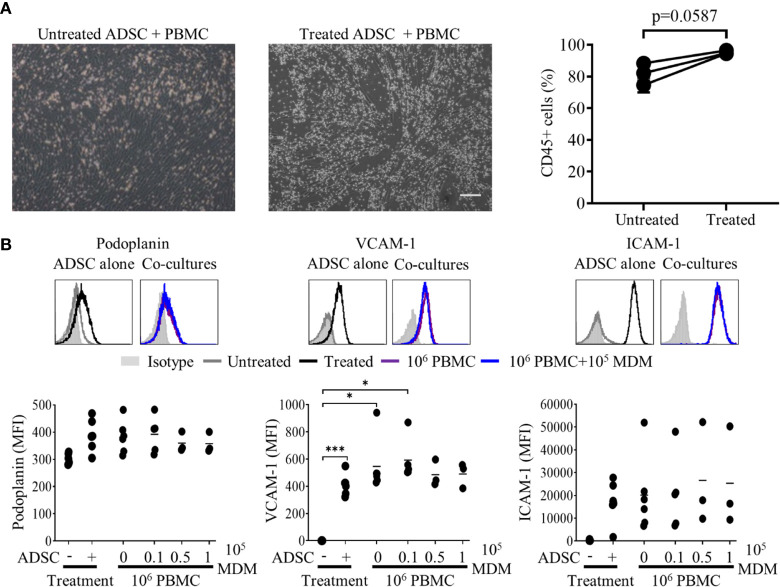
Lymphocytes interact with adipocyte derived stem cells (ADSC)-derived lymphoid fibroblasts. **(A)** Peripheral blood mononuclear cell (PBMC) were co-cultured with untreated or cytokine treated ADSC for 2 h then washed; post-wash phase contrast images are shown, scale bar, 200 µm. The graph shows the percentage of CD45+ lymphocytes recovered from wells containing untreated or cytokine-treated ADSC after 2 h of co-culture, mean +/− SD of two replicates for three independent experiments is shown. Two group comparisons were made using a paired two-tailed t-test. **(B)** Representative histogram overlays of podoplanin, vascular cell adhesion molecule 1 (VCAM-1) and intercellular adhesion molecule 1 (ICAM-1) staining at day 3 of culture for untreated and cytokine treated ADSC alone or treated ADSC co-cultured with total PBMC +/− 1 × 10^5^ autologous monocyte-derived macrophages (MDM). Isotype controls are shown as filled silver histograms. Graphs show surface expression of ICAM-1, VCAM-1 and podoplanin for each culture condition at day 3 of culture. Each solid circle represents an independent experiment, bars represent the mean. Two group comparisons were made using one-way ANOVA with Sidak correction for multiple comparisons applied, *p < 0.05, ***p < 0.001.

As an initial step in producing successful co-cultures, we assessed whether lymphocytes could interact with ADSC-derived fibroblasts. Using a previously published protocol as a basis (18), we cultured PBMC for 2 h with ADSC, previously cultured in the presence or absence of cytokines for 7 days, (differentiation confirmed by flow cytometry; **Supplementary Figure 3**), after which wells were imaged using phase contrast microscopy (**Figure 3A**) and the recovered cells analyzed by flow cytometry (right-hand panel). The proportion of harvested cells expressing CD45 was higher for cytokine treated ADSC compared to their unstimulated counterparts indicating that PBMC were better able to interact with the former. Next, we added PBMC to ADSC at a ratio of 100:1 and cultured them for 3 days in medium supplemented with cytokines, with ADSC cultured +/− cytokines serving as controls. We noted that expression of lymphoid fibroblast was similarly increased in treated ADSC +/− PBMC relative to non-treated controls (**Figure 3B**). Given the highly complex cellular make-up of the DLBCL TME (32) and the presence of TAM which has been linked with overall patient prognosis (36, 37) and response to standard of care immunochemotherapy (38, 39), we next assessed the impact of adding increasing numbers of donor-matched MDM (0.1×, 0.5×, and 10 × 10^5^ MDM), to our PBMC/ADSC co-culture system. Increasing numbers of MDM did not significantly impact the expression of lymphoid fibroblast markers on the recovered ADSC-derived fibroblasts (**Figure 3B**).

**Figure 4:**
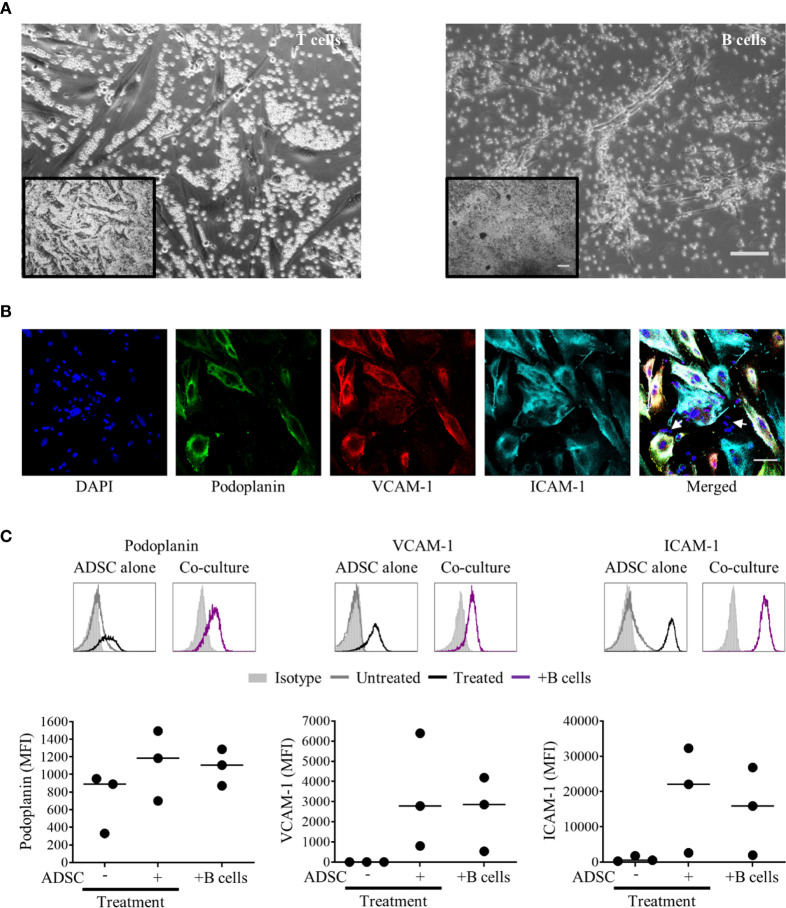
B cells rather than T cells interact with adipocyte derived stem cells (ADSC)-derived lymphoid fibroblasts. **(A)** MACS-purified T or B cells were co-cultured with ADSC in the presence of cytokines for 5 days; phase contrast images are shown. Scale bars: 200 µm, inset image, 100 µm main image. **(B)** Immunofluorescent staining of ADSC cultured on coverslips in cytokine-supplemented media in the presence of purified B cells, stained with fluorescently labeled antibodies against the indicated markers, and counterstained with DAPI; panels depict each channel alone, with the final panel showing the merged channels, with white arrows indicating nuclei of B cells in close proximity to ADSC-derived lymphoid fibroblasts, scale bar, 50 µm. **(C)** Representative histogram overlays of podoplanin, vascular cell adhesion molecule 1 (VCAM-1) and intercellular adhesion molecule 1 (ICAM-1) staining at day 6 of untreated and cytokine treated ADSC alone and cytokine treated ADSC co-cultured with MACS-purified B cells, isotype controls are shown as filled silver histograms. Graphs show surface expression of ICAM-1, VCAM-1, and podoplanin for each culture condition at day 5 of culture. Each closed circle represents an independent experiment, and bars represent mean values. Comparisons between groups were made using one-way ANOVA with Sidak correction for multiple comparisons applied, p values < 0.05 were considered significant.

Next, we isolated B or T cells from PBMC and co-cultured them with ADSC at a ratio of 100:1 for 5 days in the presence or absence of cytokines. The images show that B cells form clusters with ADSC, while T cells are restricted to inter-ADSC spaces (**Figure 4A**); IF microscopy confirmed that B cells closely associate with ADSC-derived fibroblasts (**Figure 4B**, B cells indicated by white arrows). We also assessed the impact of B cell co-culture on the ability of cytokines to modulate lymphoid stromal marker expression on ADSC. Co-culture of B cells with cytokine treated ADSC did not significantly alter the expression of lymphoid fibroblast markers compared to cytokine treated ADSC alone (**Figure 4C**). Overall, these data support the assertion that ADSC differentiated with this cytokine cocktail become B cell supportive.

### Primary DLBCL Cells Interact With ADSC-Derived Fibroblasts and Modulate Expression of Lymphoid Fibroblast Markers

**Figure 5:**
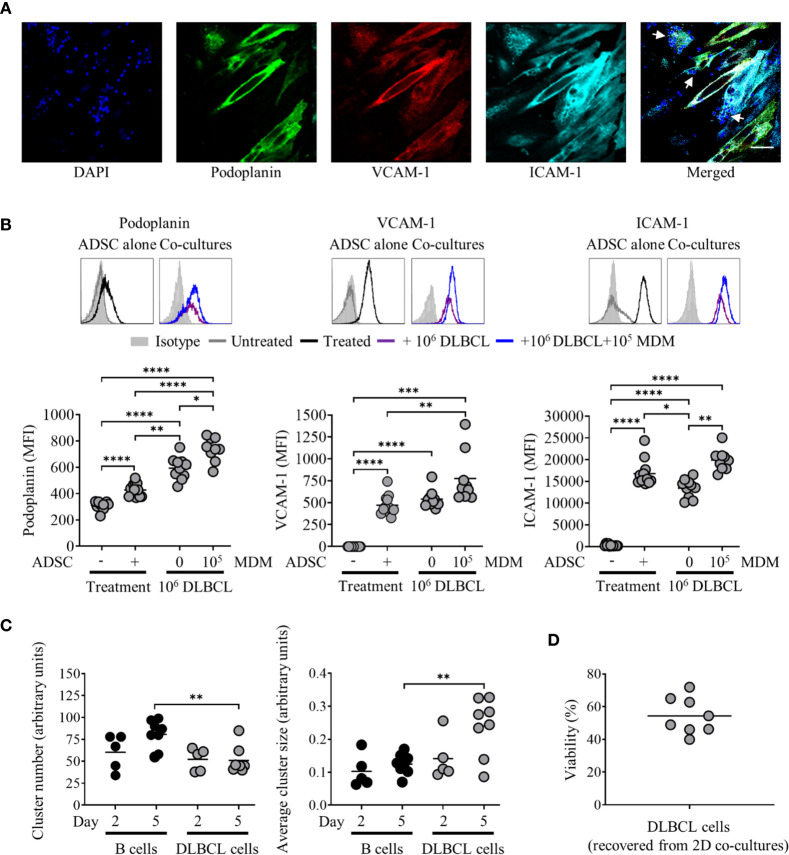
Primary diffuse large cell B cell lymphoma (DLBCL) cells interact with adipocyte derived stem cell (ADSC)-derived lymphoid fibroblasts and modulate expression of lymphoid fibroblast markers. **(A)** ADSC co-cultured with primary DLBCL on coverslips in cytokine-supplemented media were stained with fluorescently labeled antibodies against the indicated markers, and counterstained with DAPI, panels depict each channel alone. The final panel shows the merged channels, with white arrows indicating nuclei of DLBCL cells in close proximity to ADSC-derived lymphoid fibroblasts, scale bar, 50 µm. **(B)** Representative histogram overlays of podoplanin, vascular cell adhesion molecule 1 (VCAM-1) and intercellular adhesion molecule 1 (ICAM-1) staining at day 3 for untreated and cytokine treated ADSC alone or treated ADSC co-cultured with primary DLBCL cells +/− 1 × 10^5^ monocyte-derived macrophages (MDM). Isotype controls are shown as filled silver histograms. Graphs show surface expression of podoplanin, VCAM-1 and ICAM-1 for each culture condition after 3 days of culture. Each closed circle represents an independent experiment, bars represent the mean. Group comparisons were made using the one-way ANOVA test with Sidak correction for multiple comparisons, *p < 0.05, **p < 0.01, ***p < 0.001, ****p < 0.0001. **(C)** Graphs show cell cluster data calculated using ImageJ, specifically the number of cell clusters (left-hand graph) and average cluster size (right-hand graph). Pooled data from three independent experiments are shown, group comparisons were made using one-way ANOVA with Sidak correction for multiple comparisons, **p < 0.01. **(D)** Graph depicting percentage viability of primary DLBCL after 3 days of culture with ADSC-derived lymphoid fibroblasts, each symbol represents data from an independent experiment, bar represents the mean.

Given our goal to generate a model of the DLBCL TME, we next established co-cultures combining ADSC with primary DLBCL tumor cells +/− MDM. DLBCL were rested for 1 h, prior to dead cell removal. DLBCL were added at the same ratio as PBMC; in some cases, 1 × 10^5^ MDM were also added. Cells were then cultured in the presence of the cytokine cocktail for up to 8 days. DLBCL interacted with ADSC-derived fibroblasts as determined by IF (merged image panel, **Figure 5A**, DLBCL indicated by white arrows). Unlike PBMC/ADSC co-cultures, expression of lymphoid fibroblast markers differed in DLBCL/ADSC co-cultures at day 3, with podoplanin expression being significantly increased and ICAM-1 levels significantly decreased as compared to cytokine-treated ADSC alone (**Figure 5B**). Furthermore, addition of MDM to the co-culture system resulted in increased expression of all three lymphoid fibroblast markers. This pattern of expression was maintained for podoplanin after a further 5 days of culture, whereas VCAM-1 levels increased in DLBCL/ADSC co-cultures and ICAM-1 expression was similarly reduced in both DLBCL/ADSC and DLBCL/ADSC/MDM co-cultures compared to cytokine treated ADSC monocultures (**Supplementary Figure 4A**). We then analyzed bright field images of cytokine treated ADSC cultured with either B cells or DLBCL and observed that the frequency of clusters in B cell/ADSC co-cultures was significantly higher, and the average cluster size significantly lower, at day 5 than in in DLBCL/ADSC co-cultures (**Figure 5C**), indicating that fewer, larger clusters are formed in the latter, possibly due to increased migration of DLBCL toward ADSC-derived fibroblasts. Notably, cell viability was maintained at day 3 of culture (**Figure 5D**) and did not significantly change between day 3 and day 8 across all samples assessed, indicating the pro-survival impact of these co-cultures compared to DLBCL monocultures (**Supplementary Figure 4B** versus **Figure 1B**).

Given our observation of phenotypic alterations in cytokine-treated ADSC co-cultured with DLBCL and MDM, we next assessed whether the impact of cytokine withdrawal on ADSC-derived fibroblast phenotype would be lessened in this context. At day 3 co-culture, wells were either maintained in cytokine-supplemented media or switched to media alone for a further 5 days. The phenotype of ADSC-derived lymphoid fibroblasts was then assessed by flow cytometry. Removal of cytokines in the context of 2D co-cultures resulted in a reduction, but not total loss of lymphoid fibroblast marker expression on ADSC with podoplanin being the only marker significantly lower following cytokine withdrawal compared to co-cultures treated with cytokines throughout. Moreover, all three lymphoid fibroblast markers were maintained at higher levels in 2D co-culture following cytokine removal than in unstimulated ADSC (**Supplementary Figure 4C**). These data suggest that the phenotype of ADSC-derived lymphoid fibroblasts can be partially stabilized by primary DLBCL cells and MDM upon cytokine removal, indicating 2D co-culture generates, to some degree, a mutually supportive environment.

Overall, our data indicate that interactions between ADSC-derived lymphoid fibroblasts and primary DLBCL cells are qualitatively different from those observed in PBMC/ADSC co-cultures. A marked increase in podoplanin, a recognized marker of CAF, suggests interactions between ADSC-derived lymphoid fibroblasts and DLBCL drive the former toward a more CAF-like phenotype. The observed reduction in ICAM-1 expression in co-cultures suggests this adhesion molecule may be involved in the observed inter-cellular interactions, particularly as its expression decreased during culture.

### Generation of 3D Collagen Spheroid Co-Cultures

**Figure 6:**
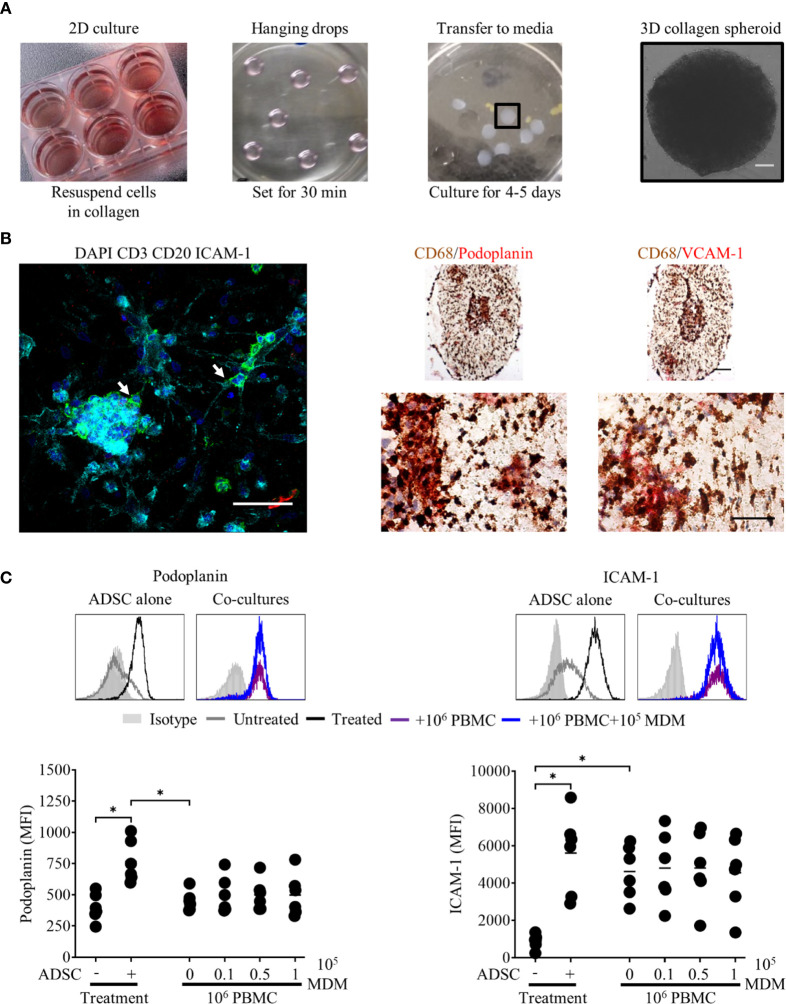
Peripheral blood mononuclear cell (PBMC) interact with adipocyte derived stem cell (ADSC)-derived fibroblasts in 3D collagen spheroid co-cultures. **(A)** Images demonstrating the culture techniques used to generate 3D collagen spheroid co-cultures. Day 3 ADSC, PBMC monocyte-derived macrophage (MDM) 2D co cultures (first panel), 12 µl droplets of cells suspended in Type-I collagen (second panel), 3D collagen spheroid co-cultures in six well-plate (third panel). The final panel depicts a phase contrast image of a 3D collagen spheroid co-culture at day 8 of culture, scale bar 200 µm. **(B)** Left-hand panel: Whole-mount staining of a 3D collagen spheroid co-culture containing ADSC (ICAM-1, cyan) and PBMC [B cells, CD20 (green), T cells, CD3 (red)], counterstained with DAPI nuclear stain (blue). White arrows indicate B cells in close association with ADSC-derived lymphoid-like fibroblasts, scale bar, 50 µm. Right-hand panel; representative double immunohistochemistry (IHC) staining of formaldehyde fixed paraffin embedded (FFPE) sections of collagen spheroid co-cultures containing ADSC-derived lymphoid fibroblasts (podoplanin and VCAM-1, red), PBMC, and MDM (CD68, brown), scale bars 100 µm and 50 µm for upper and lower images, respectively. **(C)** Representative histogram overlays of podoplanin and ICAM-1 staining of untreated and cytokine treated ADSC alone or cytokine treated ADSC co-cultured with PBMC +/− 10^5^ autologous MDM recovered from 3D collagen spheroid cultures after 8 days of culture, isotype controls are shown as filled silver histograms. Graphs show surface expression of podoplanin and ICAM-1 on lymphoid fibroblasts for each culture condition at day 8 of culture. Each closed circle represents data from an independent experiment, bars represent the mean. Group comparisons were made using the one-way ANOVA test with Sidak correction for multiple comparisons; p values < 0.05 were considered significant, *p < 0.05.

Having shown that both PBMC and DLBCL cells differentially interact with ADSC-derived lymphoid stroma in standard 2D tissue culture, we next investigated whether these interactions would be maintained and potentially enhanced in the context of 3D cultures. We chose a scaffold-based system that would allow the incorporation of the previously tested 2D co-cultures into a Type I collagen-based extracellular matrix, generating 3D spheroids that could be subsequently cultured. **Figure 6A** illustrates the steps involved. Post-culture, the recovered spheroids were subdivided and either disaggregated and analyzed by flow cytometry, directly stained for immunofluorescence, or fixed in formaldehyde prior to embedding in paraffin and sectioning, for subsequent staining by IHC.

**Figure 7:**
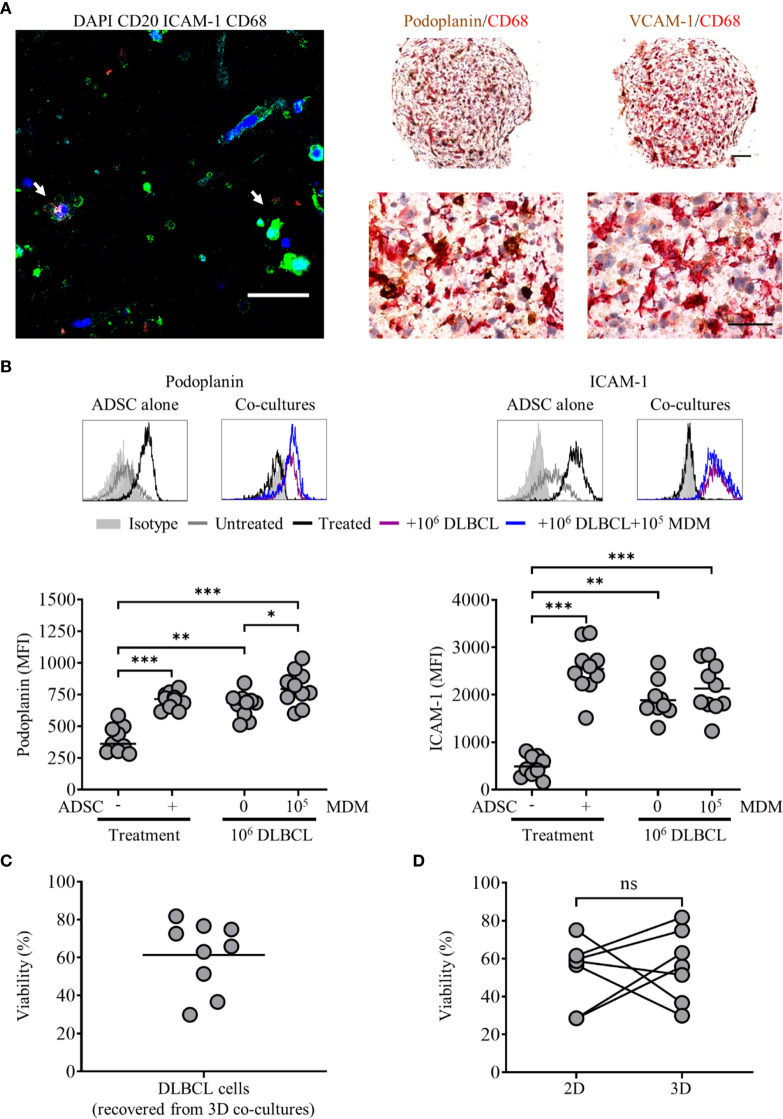
Primary diffuse large cell B cell lymphoma (DLBCL) cells interact with adipocyte derived stem cell (ADSC)-derived fibroblasts in 3D collagen spheroid co-cultures. **(A)** Left-hand panel: Whole-mount staining of a 3D collagen spheroid co-culture containing ADSC (ICAM-1, cyan), primary DLBCL cells (CD20, green), and monocyte-derived macrophages (MDM) (CD68, red), counterstained with DAPI (blue). White arrows indicate DLBCL cells in close association with ADSC-derived lymphoid-like fibroblasts and MDM, scale bar, 50 µm. Right-hand panel; representative double IHC staining of FFPE sections of 3D spheroid co-cultures containing ADSC-derived lymphoid fibroblasts (podoplanin and VCAM-1, brown), primary DLBCL cells and MDM (CD68, red), scale bars 100 µm and 50 µm for upper and lower images, respectively. **(B)** Representative histogram overlays of podoplanin and ICAM-1 staining of untreated and cytokine treated ADSC alone or treated ADSC co-cultured with primary DLBCL cells +/− 1 × 10^5^ MDM recovered from 3D collagen spheroid cultures after 8 days of culture, isotype controls are shown as filled silver histograms. Graphs show surface expression of podoplanin and ICAM-1 for each culture condition at day 8 of culture. Each closed circle represents an independent experiment, bars represent the mean. Group comparisons were made using a one-way ANOVA with Sidak correction for multiple comparisons; p values < 0.05 were considered significant, *p < 0.05, **p < 0.01, ***p < 0.001. **(C)** Viability of primary DLBCL cells recovered from 3D collagen spheroid co-cultures after 8 days of culture, each symbol represents data from an independent experiment, bars represent the mean (left-hand graph). **(D)** Comparison of the viability of primary DLBCL recovered from 2D co-cultures or 3D spheroid co-cultures cultured in parallel for 8 days (right-hand graph). Each pair of linked filled circles represents data from an independent experiment. Two group comparisons were made using a paired t-test; p values <0.05 were considered significant, ns, not significant.

3D collagen spheroid co-cultures, stained and analyzed by IF microscopy, revealed ADSC-derived lymphoid fibroblasts (ICAM-1-positive cells) and B cells (CD20 positive cells) were in close proximity to each other (**Figure 6A**, left-hand panel, white arrows). IHC of 3 µm sections generated from FFPE 3D spheroids, stained for MDM (CD68) and lymphoid fibroblast markers (podoplanin and VCAM-1) showed that these populations also interacted with each other in a 3D context (**Figure 6B**, right-hand panel). The pattern of fibroblast marker expression on ADSC-derived lymphoid fibroblasts isolated from 3D spheroid PBMC co-cultures, as determined by flow cytometry (**Figure 6C**), differed to that seen in 2D co-cultures (see **Figure 3B**). Notably we were unable to detect VCAM-1 post-disaggregation, irrespective of the anti-human VCAM-1 antibody used (**Supplementary Figure 5A**), although IHC revealed the presence of VCAM-1 positive cells in sections from cytokine-treated 3D collagen spheroid cultures (**Figures 6B** and **7A**, and **Supplementary Figure 5B**). Although podoplanin expression on ADSC-derived lymphoid fibroblasts recovered from cytokine-treated 3D collagen spheroids was significantly higher compared to those from untreated controls, its expression on ADSC-derived lymphoid fibroblasts recovered from 3D PBMC/ADSC spheroid co-cultures, irrespective of the presence of MDM, was similar to that of untreated controls. ICAM-1 expression levels on ADSC-derived lymphoid fibroblasts recovered from cytokine-treated 3D spheroids were lower than seen in their 2D counterparts, but the pattern was similar, with significantly increased expression on cells recovered from cytokine treated 3D ADSC monocultures and 3D PBMC/ADSC co-cultures (**Figure 6C**). Overall, these flow cytometry data suggest a qualitative difference in the interaction of cell populations in 3D versus 2D ADSC/PBMC co-cultures.

### Interactions Between Primary DLBCL Cells and ADSC-Derived Fibroblasts Persist in 3D Collagen Spheroids

We next applied our 3D collagen spheroid protocol to primary DLBCL cells. Fluorescence microscopy of immunostained 3D collagen spheroid co-cultures revealed ADSC-derived lymphoid fibroblasts (ICAM-1), DLBCL cells (CD20) and MDM (CD68) in close proximity to each other (**Figure 7A**, left-hand panel, white arrows). IHC corroborated the close interaction of these populations in 3D collagen spheroid co-cultures (**Figure 7A**, right-hand panels). Of note, the pattern of both podoplanin and ICAM-1 expression on ADSC-derived lymphoid fibroblasts recovered from 3D spheroid co-cultures (**Figure 7B**) was very similar to that observed in day 8 2D co-cultures (**Supplementary Figure 4**). The viability of DLBCL recovered from 3D collagen ADSC/DLBCL/MDM spheroid co-cultures was also maintained (**Figure 7C**). Moreover, there was no significant difference between the viability of cells recovered from 3D and parallel 2D co-cultures harvested at this time point (**Figure 7D**).

To assess the impact of cytokine withdrawal on ADSC-derived lymphoid fibroblast phenotype in the context of 3D spheroid co-cultures, we generated 3D DLBCL/ADSC/MDM spheroid co-cultures as previously described, and then cultured them in medium +/− cytokines for 5 days. Cultures were then stopped, and the phenotype of the recovered ADSC-derived fibroblasts assessed by flow cytometry. Unlike 2D co-cultures where cytokine removal resulted in a reduction, but not total loss of lymphoid fibroblast marker expression (**Supplementary Figure 4C**), ADSC-derived lymphoid fibroblasts recovered from 3D collagen spheroid co-cultures cultured in medium alone maintained lymphoid fibroblast marker expression at similar levels to those of cells recovered from 3D spheroids cultured in cytokine-supplemented medium (**Supplementary Figure 6A**). Furthermore, IHC of spheroids cultured in medium +/− cytokines also showed similar patterns of podoplanin and CD68 expression (**Supplementary Figure 6B**). These data indicate that the ADSC-derived lymphoid fibroblast phenotype is maintained in the 3D co-culture environment even in the absence of cytokines. Overall, these data provide further evidence that ADSC-derived lymphoid fibroblasts, DLBCL cells and MDM can interact and that, in the context of 3D collagen spheroid co-cultures, generate a self-sustainable environment. The maintenance of primary DLBCL cell viability in the context of the 3D co-culture system further supports this observation and indicates that the system could be adapted to study the impact of therapeutic agents.

### DLBCL Are Targets for Antibody Dependent Cellular Phagocytosis in 2D and 3D Models

**Figure 8:**
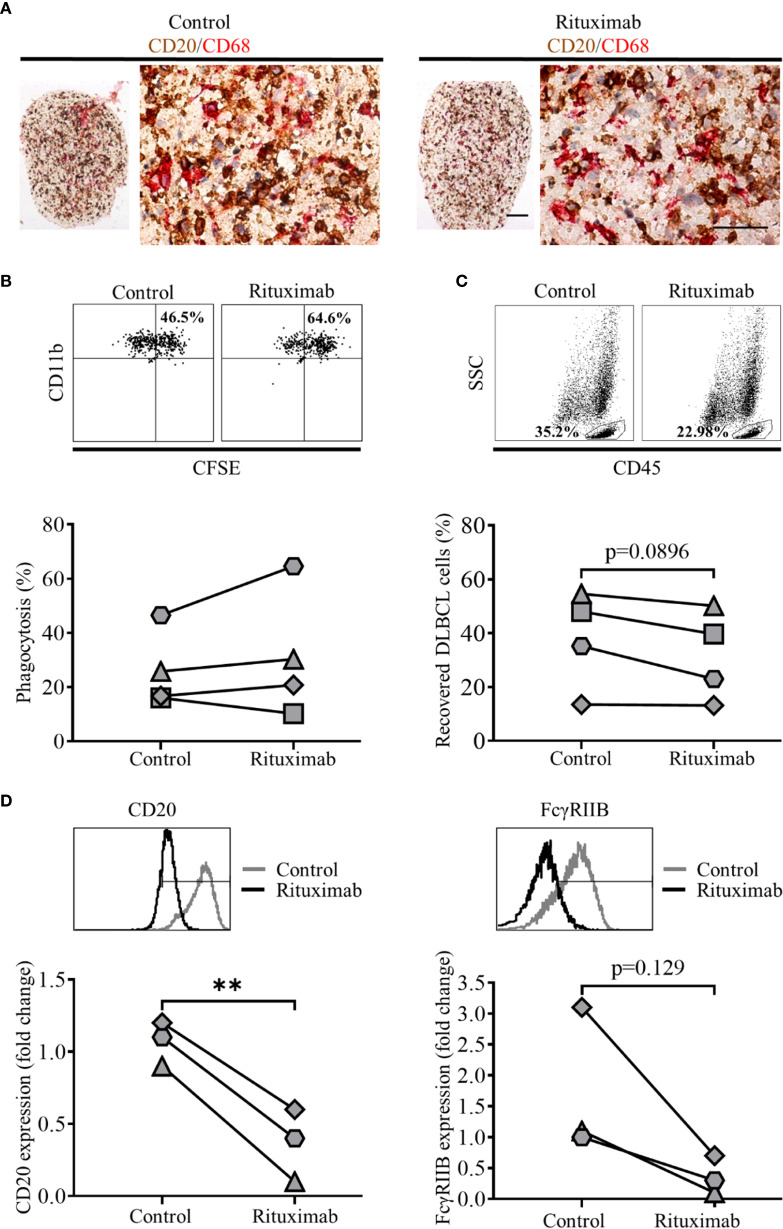
Treatment of 3D spheroid co-cultures with rituximab can induce antibody dependent cell phagocytosis (ADCP). **(A)** Representative double immunohistochemistry (IHC) staining of formaldehyde fixed paraffin embedded (FFPE) sections of 2 mg/ml collagen spheroid co-cultures containing adipocyte derived stem cell (ADSC)-derived lymphoid fibroblasts, primary diffuse large cell B cell lymphoma (DLBCL) cells (CD20, brown), and monocyte-derived macrophages (MDM) (CD68, red) cultured with 10 µg/ml of a control antibody (trastuzumab, left-hand panel) or rituximab (right-hand panel) for 44 h prior to harvest, scale bars 100 and 50 µm for upper and lower images, respectively. **(B)** Representative dot plots illustrating the percentage phagocytosis in cells recovered from 1 mg/ml collagen spheroid co-cultures treated with 10 µg/ml of control antibody (left-hand panel) or rituximab (right-hand panel) for 20 h prior to harvest; the graph shows the % phagocytosis in cells recovered from control antibody-treated and rituximab-treated 3D collagen spheroid co-cultures, each pair of linked filled circles represents data from an independent experiment. Two group comparisons were made using a paired two-tailed t test. **(C)** Representative dot plots illustrating the percentage of DLBCL recovered from 1 mg/ml collagen spheroid co-cultures treated with 10 µg/ml of control antibody (left-hand panel) or rituximab (right-hand panel) for 20 h prior to harvest; the graph shows the % of DLBCL cells recovered from control- and rituximab-treated 3D collagen spheroid co-cultures, each pair of linked filled circles represents data from an independent experiment. Two group comparisons were made using a paired two-tailed t test, p values <0.05 were considered significant. **(D)** Representative histograms showing the expression of CD20 (left-hand panel) and FcγRIIB (right-hand panel) on primary DLBCL recovered from 1 mg/ml collagen spheroid co-cultures treated with 10 ug/ml of control antibody (trastuzumab) or rituximab for 20 h prior to harvest. Graph shows the fold-change in the expression of CD20 (left-hand graph) and FcγRIIB (right-hand graph) on primary DLBCL recovered from rituximab-treated spheroid co-cultures relative to those recovered from control mAb-treated spheroids, values have been normalized relative to untreated controls. Each filled circle represents a single independent experiment. Two group comparisons were made using a paired t-test; p values < 0.05 were considered significant, **p < 0.01.

Current standard of care for many lymphomas, including DLBCL, involves anti-CD20 mAb (40), with ADCP being one of its main modes of action. Therefore, we next explored the suitability of primary DLBCL as targets for phagocytosis in our model systems, comparing conventional 2D and 3D cultures. To this end, DLBCL were labeled with CFSE, and treated with either 10 µg/ml of irrelevant control or anti-CD20 mAb (rituximab) and phagocytosis assessed in a standard 2D ADCP assay (30). In this assay rituximab opsonized DLBCL were preferentially phagocytosed compared to controls (**Supplementary Figure 7A**). Having established the suitability of DLBCL as targets for rituximab mediated ADCP, we next assessed whether we could measure this activity in our standard 3D collagen spheroid co-culture system. Thus, we generated spheroid co-cultures as described, using CFSE-labeled primary DLBCL cells; cultured them as before, with the addition 10 µg/ml of control or rituximab mAb 44 h prior to the end of culture. Cells were recovered and assessed by flow cytometry; following doublet exclusion, SSC high CD45+ cells were gated, and the proportion of CD11b+ CFSE+ cells within this population used to determine the level of phagocytosis. Only 1 of the 5 rituximab-treated spheroid co-cultures demonstrated increased ADCP, relative to their control-treated counterparts (data not shown). Nevertheless, IHC of FFPE sections from control- or rituximab-treated 3D collagen spheroid co-cultures, stained for DLBCL cells (CD20) and MDM (CD68) showed that these cell populations clearly interacted with each other (**Figure 8A**). As a next step, we assessed ADCP in spheroids generated with a lower collagen concentration (1 mg/ml) to determine whether this would generate a less rigid matrix that might facilitate higher levels of phagocytosis. We showed that ADSC recovered from cytokine-treated spheroids composed of 1 mg/ml collagen featured significantly increased levels of podoplanin and ICAM-1 compared to unstimulated controls (**Supplementary Figure 7B**).

We next assessed ADCP in these 3D spheroid co-cultures (1 mg/ml collagen) treated with mAb for the last 20 h of culture. We observed moderately increased ADCP following rituximab versus control mAb treatment in three of four experiments (**Figure 8B**), suggesting that the reduced collagen concentration had resulted in conditions more favorable for ADCP. Furthermore, we observed a reduction in the proportion of DLBCL, defined as the percentage of SSC low CD45+ single cells, recovered from rituximab-treated 1 mg/ml collagen spheroids as compared to their control-treated counterparts (**Figure 8C**).

Finally, to assess rituximab’s ability to enter collagen spheroid co-cultures and subsequently target the DLBCL cells within them, we assessed levels of two molecules expressed by DLBCL cells that interact with rituximab, its target molecule CD20 and the inhibitory FcγRIIB (CD32B) able to bind rituximab’s Fc region (30). Both CD20 and FcγRIIB staining were reduced on DLBCL recovered from rituximab-treated compared to control-treated spheroids (**Figure 8D**), confirming that Rituximab was able to access spheroids and interact with the primary DLBCL cells within them. Overall, these data suggest that 3D spheroid co-cultures, generated using 1 mg/ml collagen offer a potential platform to investigate therapeutic agents targeting either DLBCL cells, or the TME itself.

## Discussion

Our aim was to generate a model of the DLBCL-like TME with the potential to serve as a platform to assess therapies targeting this clinically relevant lymphoma. We chose not to focus on traditional 2D culture techniques, as the complex nature of the DLBCL microenvironment, confirmed by IHC in **Figure 1A**, would be difficult to recapitulate using this approach. Moreover, our observation that 98% of primary DLBCL cells died when cultured alone *in vitro* (**Figure 1B**) indicates that these cells require a culture system that effectively mimics the complex multicellular TME to support tumor cell survival *in vitro*. One way to achieve this is by using a 3D co-culture approach. Such systems have already been used as tools to study immune function *ex vivo* (41) and specific cellular constituents of the solid tumor TME, such as macrophages (42, 43), and CAF (44, 45).

Regarding lymphomas, a 3D model of FL combining lymphoma cell lines with dermal fibroblasts on a polystyrene matrix demonstrated enhancement of malignant B cell proliferative capacity compared to 2D stromal cell co-cultures (46). A cell aggregate-based system, utilizing lymphoma cell-lines, has been used to assess the efficacy of anti-CD20 mAb and Natural Killer (NK) cell-mediated ADCC (47–49). Other groups have developed models of the bone-marrow and vascular niches (50, 51) to study resistance to drug-induced apoptosis, while a recent 3D chip-based model of DLBCL, utilizing a variety of primary murine cells, enabled the *in vitro* modeling of the DLBCL TME and associated microvasculature (52). 3D Bioprinting is another technology currently being explored as a resource to recreate the tumor microenvironment, [recently reviewed by Mao et al. (53)]. Its ability to combine multiple cell types in pre-defined spatial arrays, coupled with the capacity to produce standardized 3D tumor environments lends itself to a precision medicine approach to tumor modeling. None of these models to date exclusively incorporate key primary human cell populations in a biologically relevant 3D matrix. Therefore, we elected to develop a scaffold-based 3D culture system in which cells are seeded on, or encapsulated within, natural or synthetic biomaterials (54) that mimic the ECM of solid tissues (55). This offers the flexibility of combining primary human cell populations identified as relevant for the DLBCL TME, specifically fibroblasts, and those implicated in mediating and modulating the activity of anti-CD20 mAb, macrophages, with primary tumor cells, within a Type I collagen-based 3D extracellular matrix, with the aim of recapitulating a DLBCL-like TME. We favor this approach as the encapsulation of cellular components within a 3D structure enables their recovery as single cell suspensions for downstream processing, as well as facilitating sample preparation for microscopy (FFPE for IHC, and/or whole mount staining for IF microscopy).

We confirmed that 2D culture of commercially obtained ADSC with IL-4, TNF-α and LT-α/β resulted in the generation of fibroblast-like cells, expressing markers associated with lymphoid fibroblasts (29), and that all three cytokines were not only required to drive this process (**Figures 2B, C**), but also to maintain it (**Supplementary Figure 2**). Although PBMC preferentially adhered to 2D cultures of cytokine stimulated ADSC (**Figure 3A**), addition of PBMC, either alone or together with increasing numbers of MDM, did not significantly alter the expression of lymphoid fibroblast markers on cytokine treated ADSC (**Figure 3B**). Furthermore, though we identified B cells as the lymphocyte population interacting directly with ADSC-derived lymphoid fibroblasts (**Figure 4A**), B cell/ADSC co-cultures did not alter the phenotype of the recovered ADSC-derived lymphoid fibroblasts (**Figure 4B**). In contrast, co-culturing cytokine treated ADSC with primary DLBCL cells clearly impacted the expression of podoplanin, VCAM-1, and ICAM-1 on the recovered ADSC, with addition of MDM further modulating this effect (**Figure 5B**, **Supplementary Figure 4A**), suggesting that MDM may foster unique interactions between these cells, stroma and primary DLBCL cells. The reduced ICAM-1 expression at both day 3 and 8 of 2D co-culture suggest that this adhesion molecule plays a role in mediating interactions between the three cellular constituents in this context. The observed increase in podoplanin expression was also a characteristic feature of both DLBCL/ADSC and/DLBCL/ADSC/MDM 2D co-cultures, which is notable, given the reported enrichment of the DLBCL stromal-1 gene signature in podoplanin expressing cells, including lymph node FRC (56). The viability of primary DLBCL cells was also maintained in 2D co-cultures for up to 8 days, supporting the ability of our tri-partite co-culture system to provide signals necessary to maintain DLBCL cell survival.

Our next step was to incorporate 2D co-cultures into a Type-I collagen matrix, to generate 3D spheroids (**Figure 6A**). Enzymatic digestion of these spheroids impacted upon the ability to detect adhesion molecule expression on the recovered ADSC by flow cytometry, reducing ICAM-1 MFI, and rendering VCAM-1 undetectable, indicating that these molecules likely mediate interactions between the ADSC-derived lymphoid fibroblasts and the 3D collagen scaffold. Microscopic assessment of 3D PBMC/ADSC spheroid co-cultures demonstrated that B cells and MDM maintain a close association with ADSC-derived fibroblasts in this context. Expression of lymphoid fibroblast markers on ADSC-derived fibroblasts recovered from 3D spheroid co-cultures differed to that observed for 2D co-cultures (**Figures 6C** and **3B**, respectively), suggesting that the 3D PBMC/ADSC/MDM spheroid co-cultures were qualitatively different to their 2D counterparts. 3D DLBCL/ADSC/MDM spheroid co-cultures also featured close interaction between all three cell populations in the 3D environment, with these associations comparable to those observed in clinical samples (**Figure 7A**, right-hand panels, and **Figure 1A**, respectively). Similar to 2D co-cultures, ADSC-derived fibroblasts recovered from 3D collagen spheroid co-cultures featured increased podoplanin expression and decreased ICAM-1 (**Supplementary Figure 4A** and **Figure 7B**, respectively). The viability of DLBCL cells recovered from 3D collagen spheroid co-cultures and 2D co-cultures ran in parallel, was similar suggesting that the 3D co-culture system generated an environment compatible for DLBCL cell survival (**Figures 7C, D**). Interestingly, recent data indicate that BAFF-expressing FRC promote the survival of DLBCL cells in 3D matrix gel co-cultures (14), highlighting a key support role for lymphoid fibroblasts in the DLBCL TME. However, a role for MDM in the improved survival of DLBCL in our system cannot be excluded, given the reported supportive role of monocytes in DLBCL survival and proliferation (57). Further evidence that the 3D spheroid co-culture environment featured supportive interactions between its constituents was provided by the preservation of podoplanin and ICAM-1 expression on ADSC-derived lymphoid fibroblasts recovered from 3D DLBCL/ADSC/MDM spheroid co-cultures following cytokine removal (**Supplementary Figure 6A**). This ability to compensate for the loss of extrinsic cytokine-derived signals is supported by recent data showing that co-culture of DLBCL cells with human lymph node-derived FRC increased their expression of podoplanin, a process linked to lymphoma secreted lymphotoxins and TNF-α (14).

Although our model is a simplistic representation of the *in vivo* DLBCL TME, it does include macrophages; these cells usually represent the highest proportion of inflammatory cells in the TME, sometimes as much as 50%, depending on tumor type (58). They are also key effector cells involved in delivering anti-tumor effects through mAb therapy.

Using rituximab as a model therapeutic agent in our 3D co-culture system, we noted increased phagocytosis in rituximab vs control-treated spheroids in three of four experiments (**Figure 8B**). Furthermore, there was a reduction in the percentage of DLBCL cells recovered from the rituximab-treated spheroids compared to their control-treated counterparts (**Figure 8C**).

The primary DLBCL cells we used in all our assays, from a single donor, expressed FcγRIIB (**Figure 8D**), a receptor known to bind the Fc portion of rituximab at the lymphoma cell surface resulting in its internalization and/or trogocytosis (30). The observation of down-regulation of both CD20 and FcγRIIB in rituximab-treated cultures (**Figure 8D**) suggests that this process may underlie the observed low levels of ADCP. It must be noted that CD20 expression was assessed using an in-house labeled rituximab, so we cannot exclude a competition effect underlying this reduction in CD20 expression. However, the concurrent reduction in CD19 expression on these cells (data not shown) suggests that CD20 and its associated B cell receptor components such as CD19 (59) are being internalized. Blocking of FcγRIIB could potentially overcome this issue (60). Alternatively using a type II anti-CD20 antibody such as obinutuzumab, which is not subject to such marked internalization (26, 30) could result in improved ADCP in our system.

The levels of rituximab mediated ADCP observed in our 3D spheroid co-cultures were low, suggesting that they are potentially antagonistic to ADCP, given the activity of the MDM in 2D cultures (**Supplementary Figure 7A**). This raises the possibility that a similar environment may exist in the DLBCL TME *in vivo*. The limited ADCP that we observed does, however, provide the opportunity to test agents that repolarize macrophages to a more phagocytic phenotype. Ligands that target pattern recognition receptors expressed by macrophages such as Pam3CSK4, as well as STING agonists, have been shown to enhance ADCP by altering the relative expression of activatory and inhibitory FcγRs (A:I ratio) (24) and so such reagents and strategies could be tested in follow on studies, and extended to include novel therapeutic approaches directly targeting other TME constituents.

Overall, we demonstrated that 3D collagen spheroids incorporating ADSC-derived fibroblasts co-cultured with primary DLBCL cells and MDM can be generated and are amenable to subsequent manipulation and downstream analysis by flow cytometry and microscopy. These spheroids recreate the spatial relationship of the cell populations observed in the DLBCL TME, and, to some degree, the inter cell communication that occurs within it, as evidenced by the maintenance of DLBCL viability and impact upon ADSC-derived lymphoid fibroblast phenotype. Although our current system is labor-intensive, the application of microfluidic technologies could help ameliorate this issue. Furthermore, the ability to combine multiple cell types in a 3D scaffold means it has the capacity to be modified to study other lymphomas and/or solid tumors. The modular nature of system further lends itself to personalized medicine; for example, Majudmer et al. (61) recently developed an *ex vivo* system comprising tumor sections cultured on grade-matched tumor matrix in the presence of autologous serum. Similarly, our system could be adapted to incorporate patient-derived CAF and/or TAM with the corresponding primary tumor cells in a 3D scaffold based upon the matrix within the patient’s tumor. Regardless of the precise format, it is clear that more sophisticated model systems are required to address the complexities of cancer. Hopefully such systems will elicit more effective therapies in the future.

## Data Availability Statement

The raw data supporting the conclusions of this article will be made available by the authors without undue reservation.

## Ethics Statement

Ethical approval for the use of human tumor samples was obtained by Southampton University Hospitals NHS Trust from Southampton and South West Hampshire Research Ethics Committee. Informed consent was provided in accordance with the Declaration of Helsinki. DLBCL samples were obtained from Human Tissue Authority Licensed University of Southampton, Cancer Sciences Unit Tissue Bank. Use of human blood samples was approved by the East of Scotland Research ethics service. Blood donors provided their informed consent to participate in research studies.

## Author Contributions 

RF, PN, ET performed the experiments. RF, PN, BG, EH, MCC, SB, and MSC designed the experiments. RF, PN, MAK, SB, and MSC analysed the data. RF, SB, and MSC wrote the manuscript. All authors contributed to the article and approved the submitted version.

## Funding

This work was supported by Bloodwise (Award number: 12050) and CRUK programme grants awarded to MC and SB (Award number: 24721), CRUK ECMC grant (Award number: A25171) and CRUK centre grant (Award number: A25139).

## Conflict of Interest

MSC is a retained consultant for BioInvent International and has performed educational and advisory roles for Baxalta, Boehringer Ingleheim and Merck GdA. He has received research funding from Roche, Gilead, iTeos, UCB, Bioinvent International and GSK. SB has received institutional support from BioInvent for grants and patents.

The remaining authors declare that the research was conducted in the absence of any commercial or financial relationships that could be construed as a potential conflict of interest.
