# Supplementary material for: Developing a 3D B Cell Lymphoma Culture System to Model Antibody Therapy
Source: Front Immunol. 2021 Feb 8;11:605231. doi: 10.3389/fimmu.2020.605231 (PMC7897703; doi:10.3389/fimmu.2020.605231)
Supplement: Supplementary file 1 [file Presentation_1.pptx]

## Slide 1
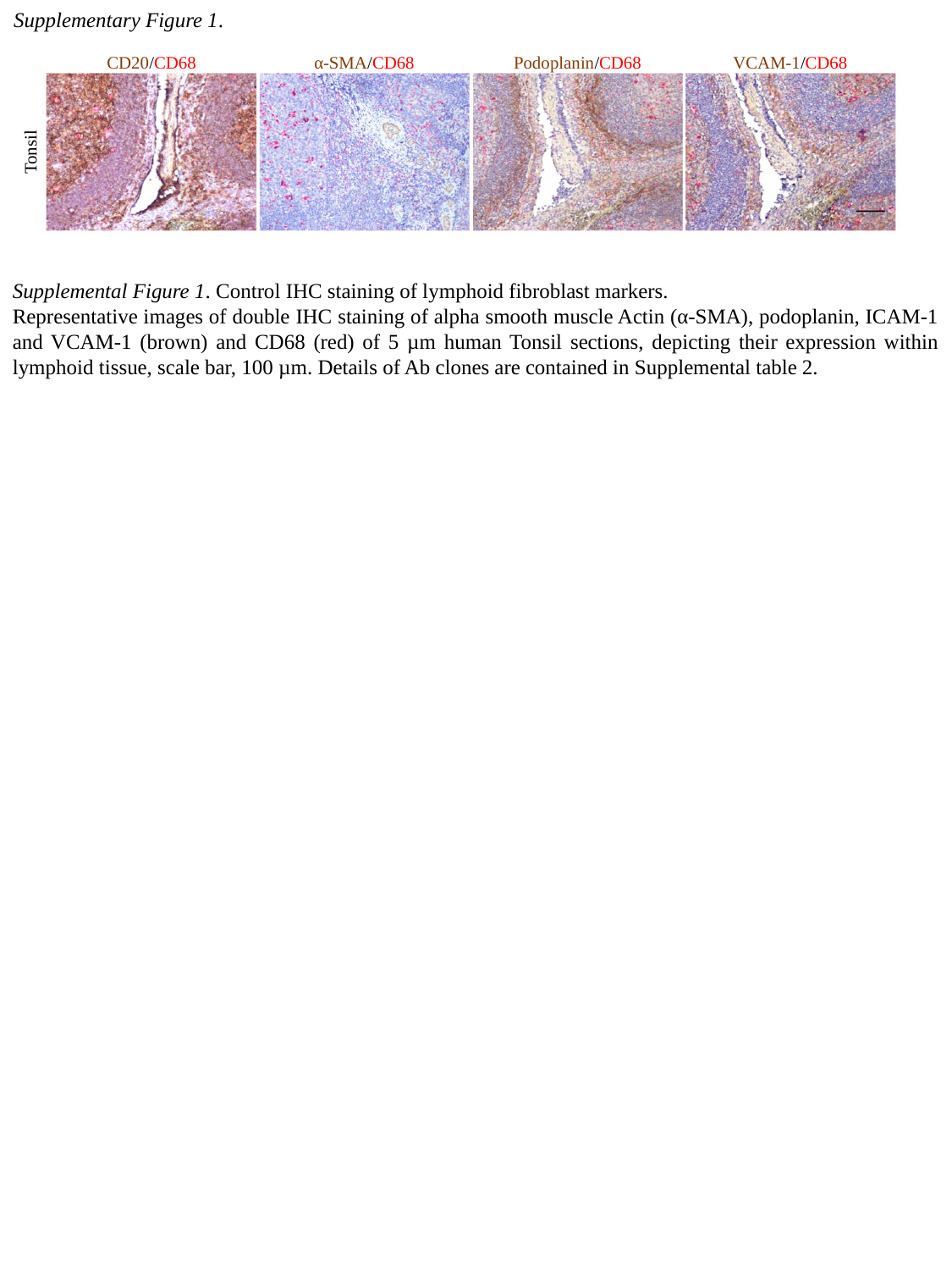

Supplementary Figure 1.
CD20/CD68
α-SMA/CD68
Podoplanin/CD68
VCAM-1/CD68
Tonsil
Supplemental Figure 1. Control IHC staining of lymphoid fibroblast markers.
Representative images of double IHC staining of alpha smooth muscle Actin (α-SMA), podoplanin, ICAM-1 and VCAM-1 (brown) and CD68 (red) of 5 µm human Tonsil sections, depicting their expression within lymphoid tissue, scale bar, 100 µm. Details of Ab clones are contained in Supplemental table 2.

## Slide 2
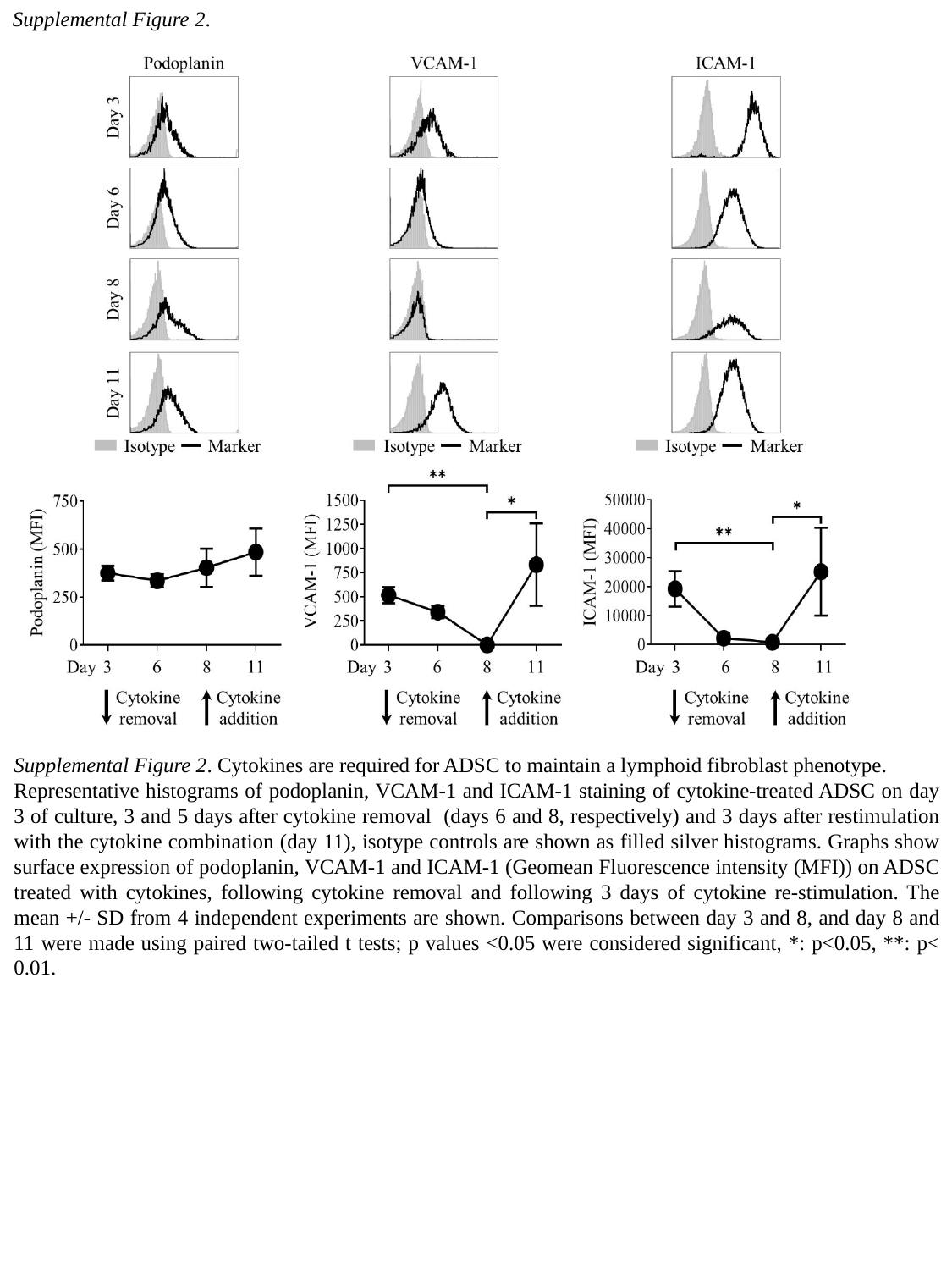

Supplemental Figure 2.
Supplemental Figure 2. Cytokines are required for ADSC to maintain a lymphoid fibroblast phenotype.
Representative histograms of podoplanin, VCAM-1 and ICAM-1 staining of cytokine-treated ADSC on day 3 of culture, 3 and 5 days after cytokine removal (days 6 and 8, respectively) and 3 days after restimulation with the cytokine combination (day 11), isotype controls are shown as filled silver histograms. Graphs show surface expression of podoplanin, VCAM-1 and ICAM-1 (Geomean Fluorescence intensity (MFI)) on ADSC treated with cytokines, following cytokine removal and following 3 days of cytokine re-stimulation. The mean +/- SD from 4 independent experiments are shown. Comparisons between day 3 and 8, and day 8 and 11 were made using paired two-tailed t tests; p values <0.05 were considered significant, *: p<0.05, **: p< 0.01.

## Slide 3
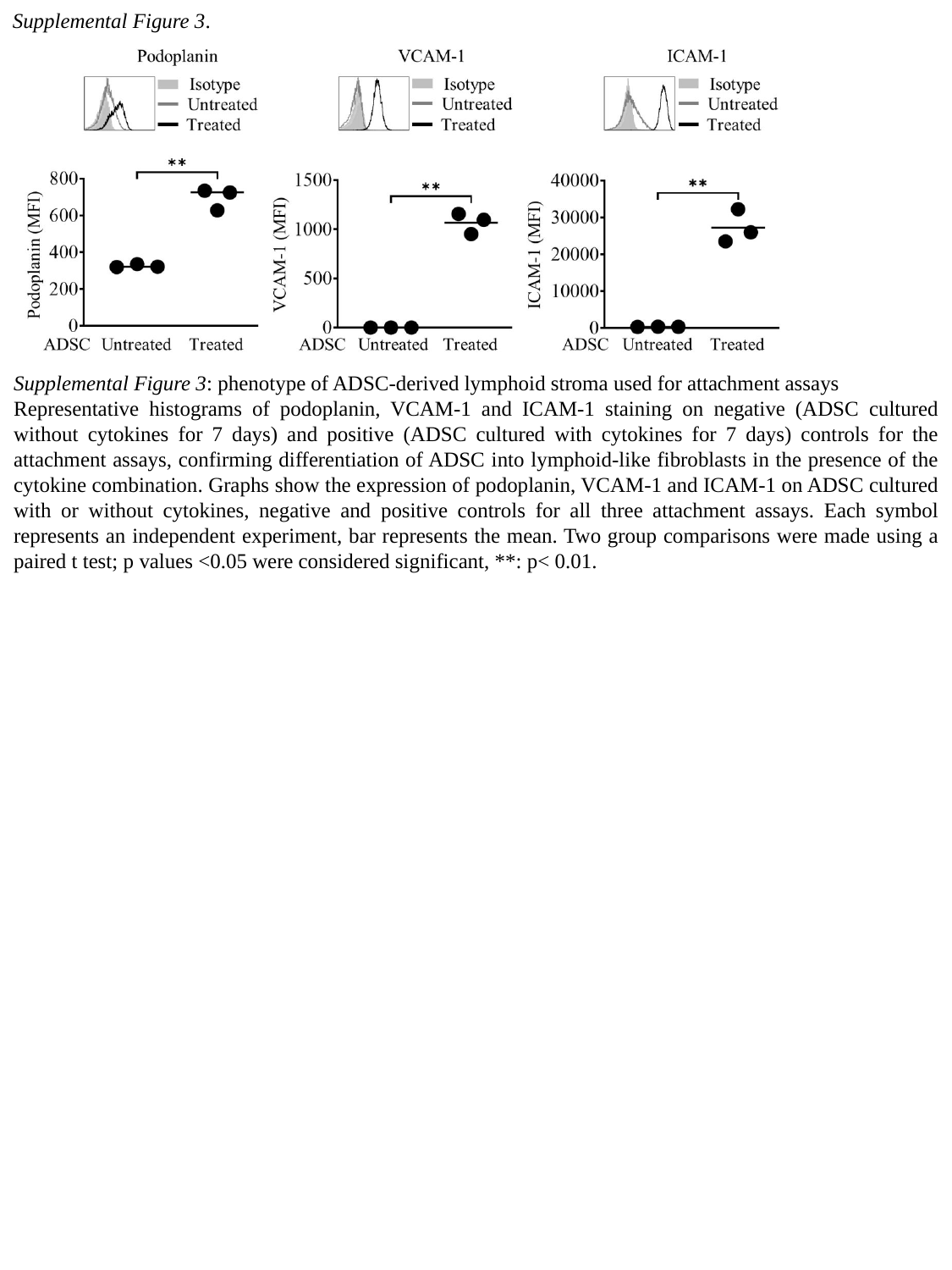

Supplemental Figure 3.
Supplemental Figure 3: phenotype of ADSC-derived lymphoid stroma used for attachment assays
Representative histograms of podoplanin, VCAM-1 and ICAM-1 staining on negative (ADSC cultured without cytokines for 7 days) and positive (ADSC cultured with cytokines for 7 days) controls for the attachment assays, confirming differentiation of ADSC into lymphoid-like fibroblasts in the presence of the cytokine combination. Graphs show the expression of podoplanin, VCAM-1 and ICAM-1 on ADSC cultured with or without cytokines, negative and positive controls for all three attachment assays. Each symbol represents an independent experiment, bar represents the mean. Two group comparisons were made using a paired t test; p values <0.05 were considered significant, **: p< 0.01.

## Slide 4
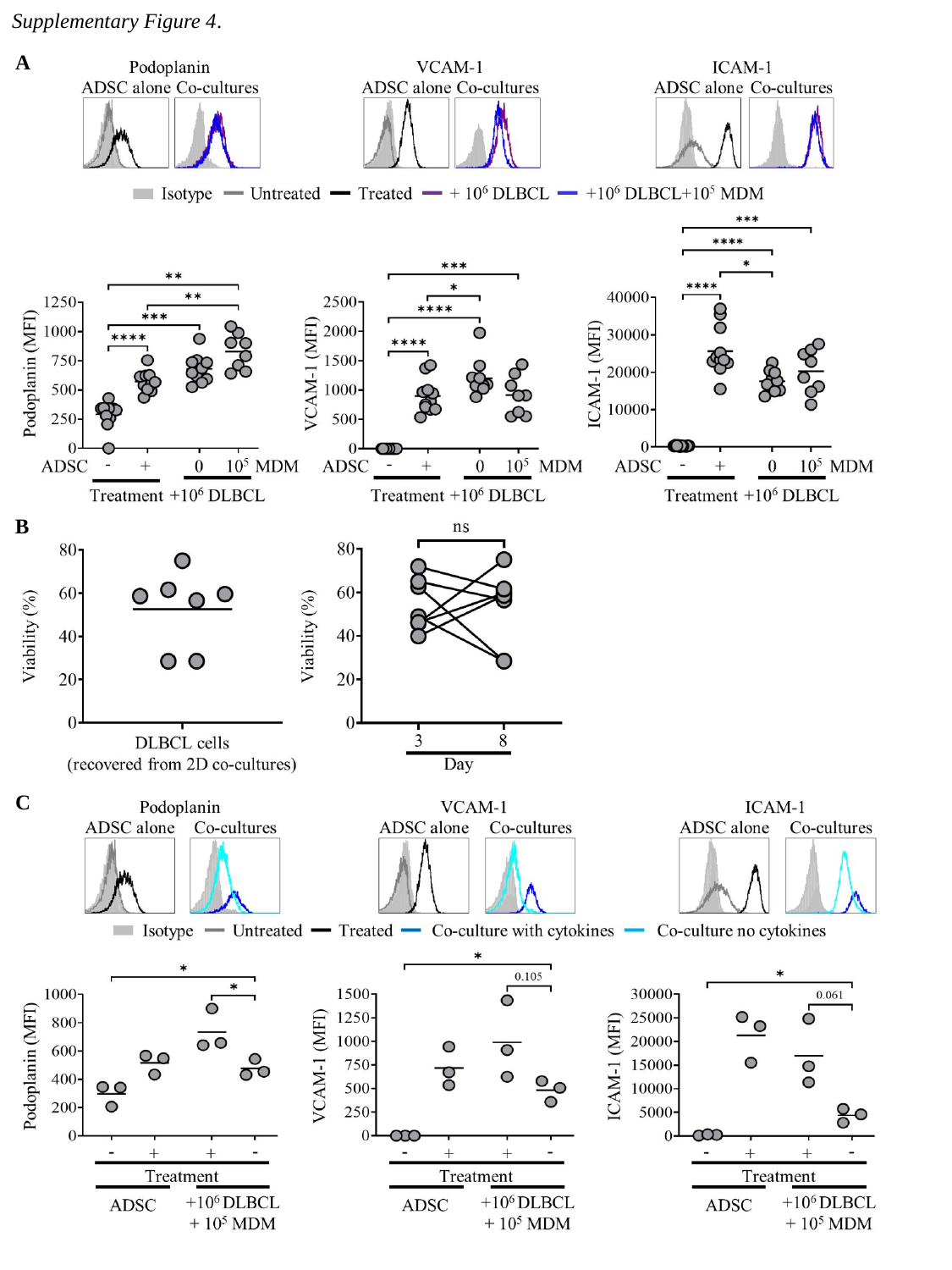

Supplementary Figure 4.
A
B
C

## Slide 5
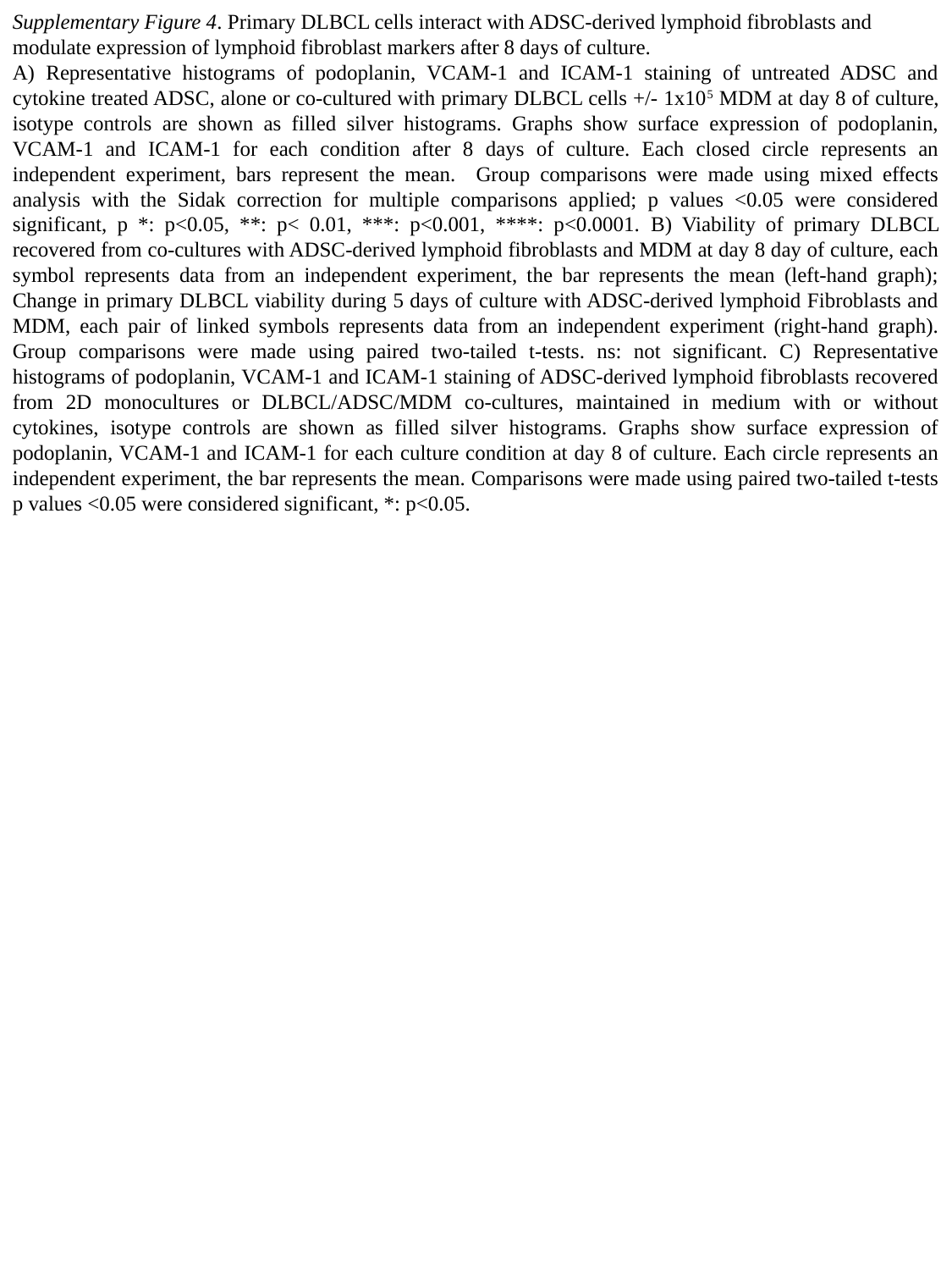

Supplementary Figure 4. Primary DLBCL cells interact with ADSC-derived lymphoid fibroblasts and modulate expression of lymphoid fibroblast markers after 8 days of culture.
A) Representative histograms of podoplanin, VCAM-1 and ICAM-1 staining of untreated ADSC and cytokine treated ADSC, alone or co-cultured with primary DLBCL cells +/- 1x10^5^ MDM at day 8 of culture, isotype controls are shown as filled silver histograms. Graphs show surface expression of podoplanin, VCAM-1 and ICAM-1 for each condition after 8 days of culture. Each closed circle represents an independent experiment, bars represent the mean. Group comparisons were made using mixed effects analysis with the Sidak correction for multiple comparisons applied; p values <0.05 were considered significant, p *: p<0.05, **: p< 0.01, ***: p<0.001, ****: p<0.0001. B) Viability of primary DLBCL recovered from co-cultures with ADSC-derived lymphoid fibroblasts and MDM at day 8 day of culture, each symbol represents data from an independent experiment, the bar represents the mean (left-hand graph); Change in primary DLBCL viability during 5 days of culture with ADSC-derived lymphoid Fibroblasts and MDM, each pair of linked symbols represents data from an independent experiment (right-hand graph). Group comparisons were made using paired two-tailed t-tests. ns: not significant. C) Representative histograms of podoplanin, VCAM-1 and ICAM-1 staining of ADSC-derived lymphoid fibroblasts recovered from 2D monocultures or DLBCL/ADSC/MDM co-cultures, maintained in medium with or without cytokines, isotype controls are shown as filled silver histograms. Graphs show surface expression of podoplanin, VCAM-1 and ICAM-1 for each culture condition at day 8 of culture. Each circle represents an independent experiment, the bar represents the mean. Comparisons were made using paired two-tailed t-tests p values <0.05 were considered significant, *: p<0.05.

## Slide 6
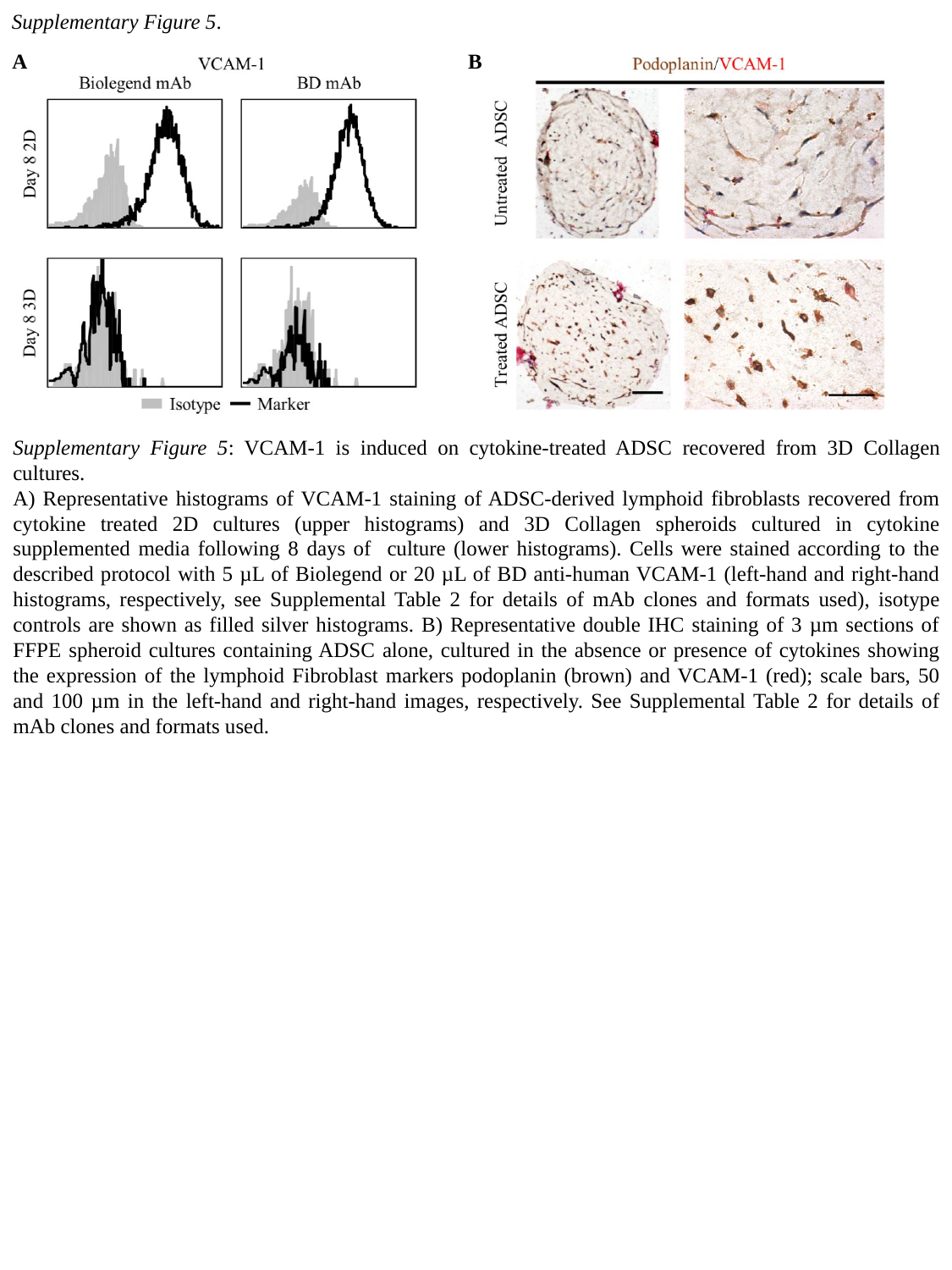

Supplementary Figure 5.
A
B
Supplementary Figure 5: VCAM-1 is induced on cytokine-treated ADSC recovered from 3D Collagen cultures.
A) Representative histograms of VCAM-1 staining of ADSC-derived lymphoid fibroblasts recovered from cytokine treated 2D cultures (upper histograms) and 3D Collagen spheroids cultured in cytokine supplemented media following 8 days of culture (lower histograms). Cells were stained according to the described protocol with 5 µL of Biolegend or 20 µL of BD anti-human VCAM-1 (left-hand and right-hand histograms, respectively, see Supplemental Table 2 for details of mAb clones and formats used), isotype controls are shown as filled silver histograms. B) Representative double IHC staining of 3 µm sections of FFPE spheroid cultures containing ADSC alone, cultured in the absence or presence of cytokines showing the expression of the lymphoid Fibroblast markers podoplanin (brown) and VCAM-1 (red); scale bars, 50 and 100 µm in the left-hand and right-hand images, respectively. See Supplemental Table 2 for details of mAb clones and formats used.

## Slide 7
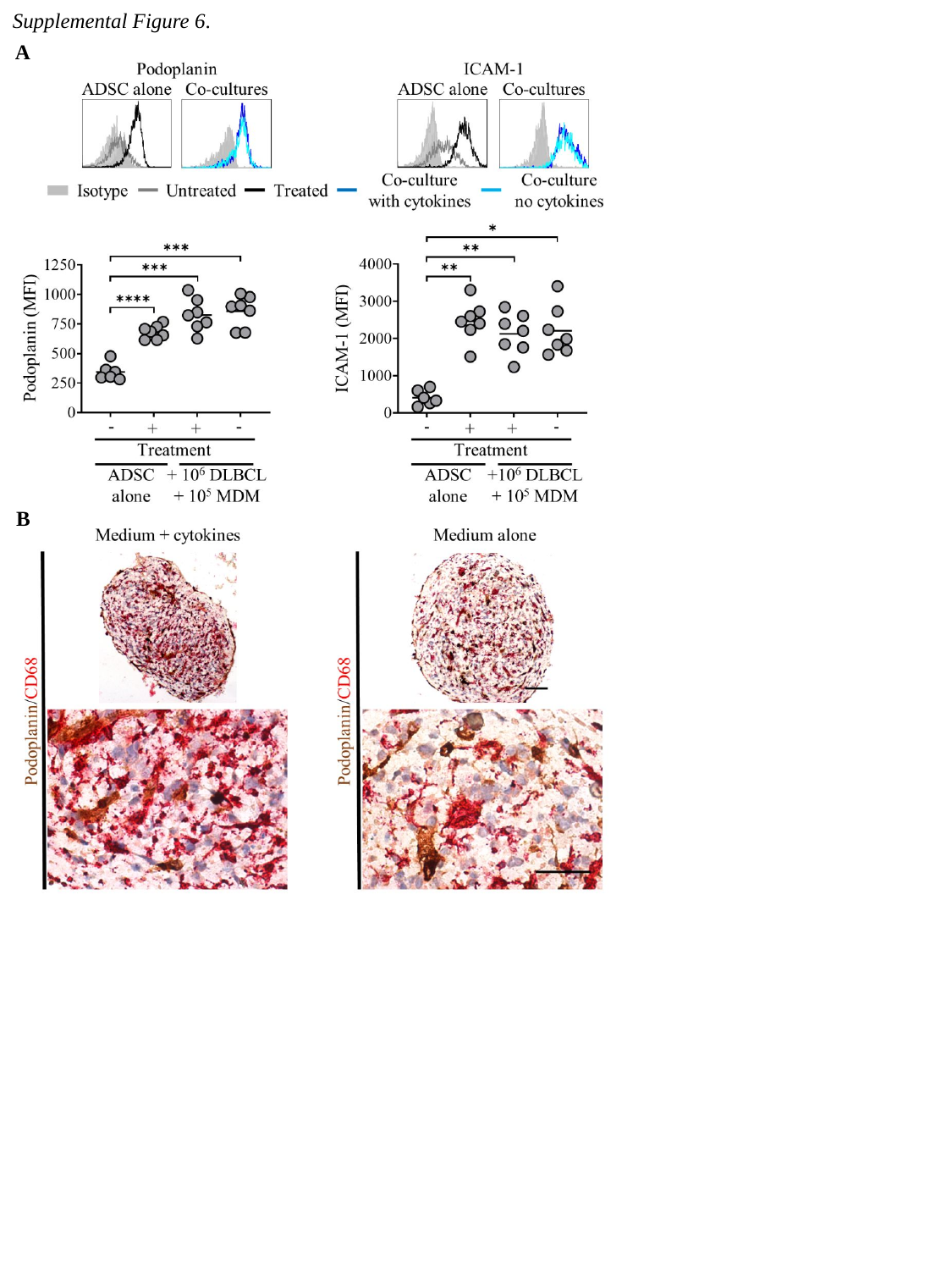

Supplemental Figure 6.
A
B

## Slide 8
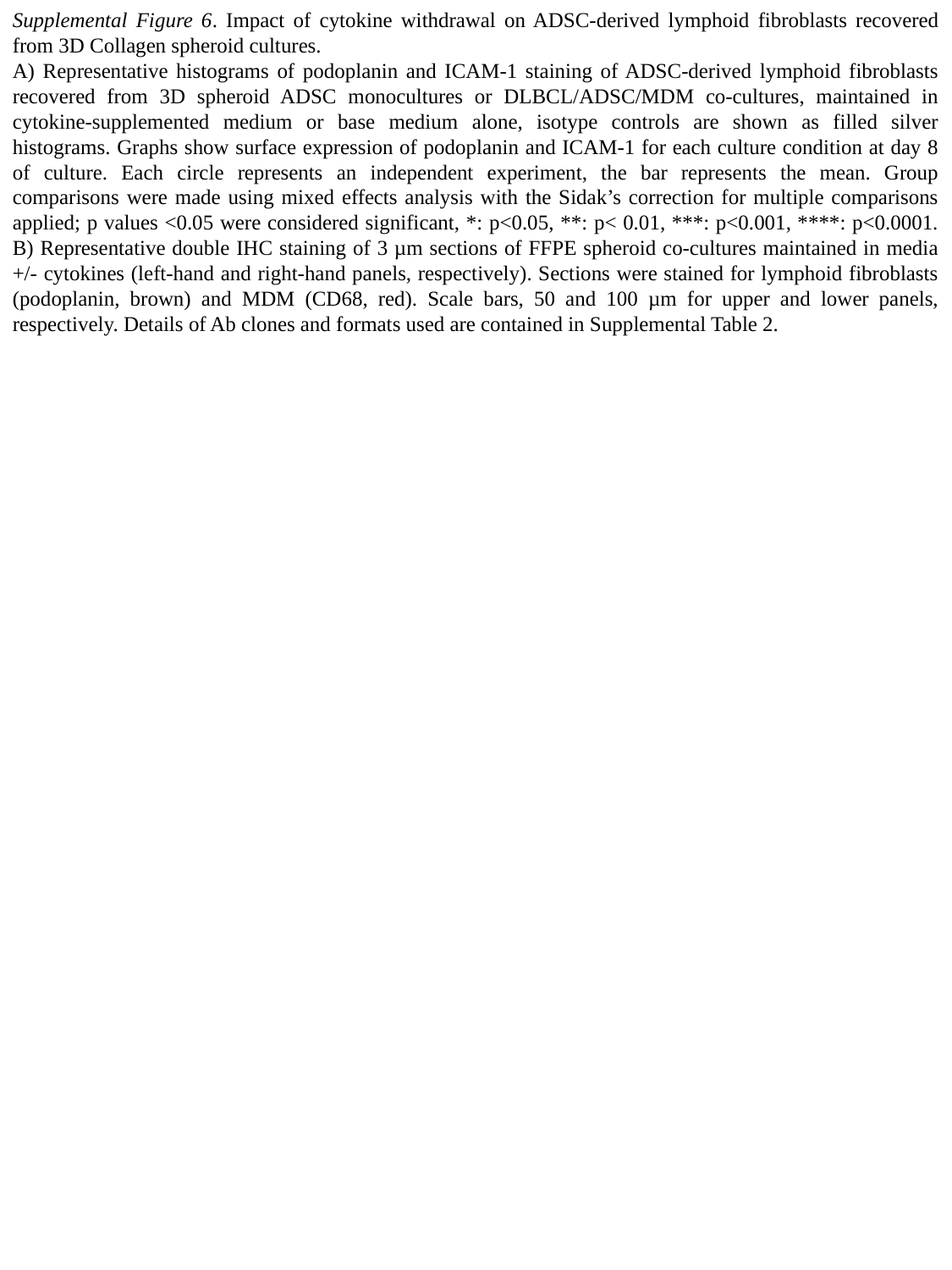

Supplemental Figure 6. Impact of cytokine withdrawal on ADSC-derived lymphoid fibroblasts recovered from 3D Collagen spheroid cultures.
A) Representative histograms of podoplanin and ICAM-1 staining of ADSC-derived lymphoid fibroblasts recovered from 3D spheroid ADSC monocultures or DLBCL/ADSC/MDM co-cultures, maintained in cytokine-supplemented medium or base medium alone, isotype controls are shown as filled silver histograms. Graphs show surface expression of podoplanin and ICAM-1 for each culture condition at day 8 of culture. Each circle represents an independent experiment, the bar represents the mean. Group comparisons were made using mixed effects analysis with the Sidak’s correction for multiple comparisons applied; p values <0.05 were considered significant, *: p<0.05, **: p< 0.01, ***: p<0.001, ****: p<0.0001. B) Representative double IHC staining of 3 µm sections of FFPE spheroid co-cultures maintained in media +/- cytokines (left-hand and right-hand panels, respectively). Sections were stained for lymphoid fibroblasts (podoplanin, brown) and MDM (CD68, red). Scale bars, 50 and 100 µm for upper and lower panels, respectively. Details of Ab clones and formats used are contained in Supplemental Table 2.

## Slide 9
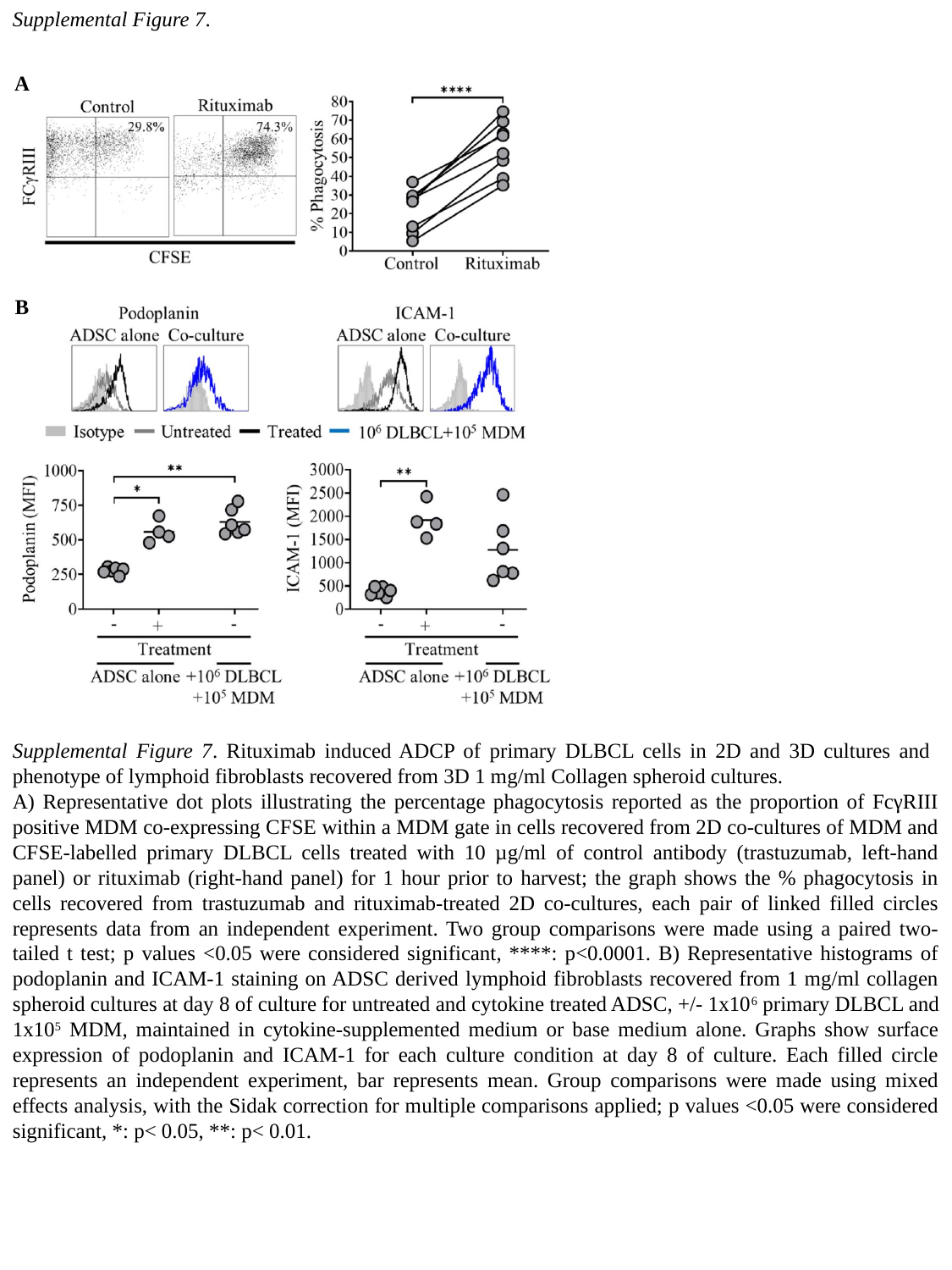

Supplemental Figure 7.
A
B
Supplemental Figure 7. Rituximab induced ADCP of primary DLBCL cells in 2D and 3D cultures and phenotype of lymphoid fibroblasts recovered from 3D 1 mg/ml Collagen spheroid cultures.
A) Representative dot plots illustrating the percentage phagocytosis reported as the proportion of FcγRIII positive MDM co-expressing CFSE within a MDM gate in cells recovered from 2D co-cultures of MDM and CFSE-labelled primary DLBCL cells treated with 10 µg/ml of control antibody (trastuzumab, left-hand panel) or rituximab (right-hand panel) for 1 hour prior to harvest; the graph shows the % phagocytosis in cells recovered from trastuzumab and rituximab-treated 2D co-cultures, each pair of linked filled circles represents data from an independent experiment. Two group comparisons were made using a paired two-tailed t test; p values <0.05 were considered significant, ****: p<0.0001. B) Representative histograms of podoplanin and ICAM-1 staining on ADSC derived lymphoid fibroblasts recovered from 1 mg/ml collagen spheroid cultures at day 8 of culture for untreated and cytokine treated ADSC, +/- 1x10^6^ primary DLBCL and 1x10^5^ MDM, maintained in cytokine-supplemented medium or base medium alone. Graphs show surface expression of podoplanin and ICAM-1 for each culture condition at day 8 of culture. Each filled circle represents an independent experiment, bar represents mean. Group comparisons were made using mixed effects analysis, with the Sidak correction for multiple comparisons applied; p values <0.05 were considered significant, *: p< 0.05, **: p< 0.01.
