# Supplementary material for: Developing a 3D B Cell Lymphoma Culture System to Model Antibody Therapy
Source: Front Immunol. 2021 Feb 8;11:605231. doi: 10.3389/fimmu.2020.605231 (PMC7897703; doi:10.3389/fimmu.2020.605231)
Supplement: Supplementary file 2 [file Table_1.docx]

|  | | | |
| --- | --- | --- | --- |
| Supplemental Table 1.  ADSC seeding density and required volumes of TrypLE Express for cell detachment in different tissue culture plates and flasks | | | |
| Culture vessel | TrypLE Express (ml) | Seeding density (ADSC/cm^2^) |  |
| 6 well-plate | 1.0 | 50,000 |  |
| 12 well-plate | 0.5 | 20, 000 |  |
| T25^a^ flask | 2.0 | 125,000 |  |
| T75^b^ flask | 3.0 | 375,000 |  |
| T125^c^ rlask | 4.0 | 875,000 |  |
| ^a^: 25 cm^2^, ^b^: 75 cm^2^, ^c^: 125 cm^2^. | | | |
|  | | | |
