# Supplementary material for: Developing a 3D B Cell Lymphoma Culture System to Model Antibody Therapy
Source: Front Immunol. 2021 Feb 8;11:605231. doi: 10.3389/fimmu.2020.605231 (PMC7897703; doi:10.3389/fimmu.2020.605231)
Supplement: Supplementary file 3 [file Table_2.docx]

| **Supplementary Table 2. Primary and secondary antibodies** | | | | | |
| --- | --- | --- | --- | --- | --- |
| **Antibody** | | **Clone** | | **Flurochrome** | **Supplier** |
| **Flow cytometry, isotype controls** | | | | | |
| Murine IgG1 | MOPC-21 | | PE, PerCP, APC, Pacific Blue | | Biolegend, UK |
| Rat IgG2a | KT3 | | Labelled in house  with Alexa Flour® 488/FITC | | In house |
| Fab’2 | db7/12 | | Labelled in house  with APC/Alexa Flour® 647 | | In house |
| **Flow cytometry, lymphoid fibroblast markers** | | | | | |
| podoplanin | NC-08 | | Alexa Flour® 488 | | Biolegend, UK |
| ICAM-1 (CD54) | HCD54 | | Alexa Flour® 647 | | Biolegend, UK |
| VCAM-1 (CD106) | STA | | PE | | Biolegend, UK |
| VCAM-1 (CD106) | 51-10C9 | | PE | | BD, UK |
| **Flow cytometry, pan-leucocyte marker** | | | | | |
| CD45 | HI30 | | Pacific Blue | | Biolegend, UK |
| **Flow cytometry, lymphocyte markers** | | | | | |
| CD3 | SK7 | | APC, PerCP | | Biolegend, UK |
| CD11b | ICRF44 | | PE, PerCP | | Biolegend, UK |
| CD19 | HIB19 | | PE, APC | | Biolegend, UK |
| CD20 | Clinical grade rituximab | | Labelled in house  with Alexa Flour® 488/FITC^a^ | | Biolegend, UK |
| **Flow cytometry, myeloid markers** | | | | | |
| CD11b | ICRF44 | | PE, PerCP | | Biolegend, UK |
| CD14 | HCD14 | | PerCP | | Biolegend, UK |
| **Flow cytometry, Fcγ receptors (FcγR)** | | | | | |
| FcγRI (CD16) | 3G8 | | Labelled in house  with APC^b^/Alexa Flour® 647^c^ | | Biovent International,  Norway |
| FcγRIIB (CD32B) | 7C07 | | Labelled in house  with APC/Alexa Flour® 647 | | Biovent International,  Norway |
| **Immunohistochemistry** | | | | | |
| alpha smooth muscle action  (α-SMA) | Rabbit polyclonal | | unconjugated | | Abcam, UK |
| ICAM-1 (CD54) | Rabbit monoclonal, EPR4776 | | unconjugated | | Abcam, UK |
| VCAM-1 (CD106) | Rabbit, EPR5047 | | unconjugated | | Abcam, UK |
| CD20 | Mouse monoclonal, L26 | | unconjugated | | DAKO, UK |
| CD68 | Mouse monoclonal, PG-M1 | | unconjugated | | DAKO, UK |
| **Immunofluorescence, primary antibodies** | | | | | |
| CD3 | Mouse monoclonal, HIT3α | | unconjugated | | BD, UK |
| CD20 | Rabbit polyclonal | | unconjugated | | Thermofisher, UK |
| CD68 | Mouse monoclonal, PG-M1 | | unconjugated | | DAKO, UK |
| podoplanin | NC-08 | | Alexa Flour® 488**^c^** | | Biolegend, UK |
| ICAM-1 (CD54) | Mouse monoclonal, HCD54 | | Alexa Flour® 647**^d^** | | Biolegend, UK |
| VCAM-1 (CD106) | Rabbit monoclonal, EPR5047 | | unconjugated | | Abcam, UK |
| **Immunofluorescence, secondary antibodies** | | | | | |
| goat anti-mouse | Goat polyclonal | | Alexa Flour® 568 | | Molecular probes,  Invitrogen, UK |
| goat anti-rabbit | Goat polyclonal | | Alexa Flour® 488 | | Molecular probes,  Invitrogen, UK |
| goat anti-rat | Goat polyclonal | | Alexa Flour® 488 | | Molecular probes,  Invitrogen, UK |
| **Therapeutic antibodies^f^** | | | | | |
| rituximab | Therapeutic antibody | | unconjugated | | UHS,  Oncology pharmacy |
| trastuzumab | Therapeutic antibody | | unconjugated | | UHS,  Oncology pharmacy |
| ^a^: Molecular probes Alexa 488 labelling kit (Thermofisher, UK), ^b^: in-house protocol using APC (Europa Bioproducts),  ^c^: Molecular probes Alexa 647 labelling kit (Thermofisher, UK), ^d^: used in conjunction with a Alexa Flour® 488 goat anti-rat secondary mAb, ^e^: no secondary antibody required, ^f^: used at 10 µg/mL in 2D and 3D ADCP assays. | | | | | |
